# Mutations of the brassinosteroid biosynthesis gene *HvDWARF5* enable balance between semi‐dwarfism and maintenance of grain size in barley

**DOI:** 10.1111/ppl.70179

**Published:** 2025-03-24

**Authors:** Karolina Zolkiewicz, Jana Oklestkova, Beata Chmielewska, Damian Gruszka

**Affiliations:** ^1^ Institute of Biology, Biotechnology and Environmental Protection, Faculty of Natural Sciences University of Silesia Katowice Poland; ^2^ Laboratory of Growth Regulators, Faculty of Science, Centre of the Region Haná for Biotechnological and Agricultural Research, Institute of Experimental Botany Czech Academy of Sciences, Palacký University Olomouc Czechia

## Abstract

Brassinosteroids (BRs) are phytohormones which regulate various developmental processes in plants. They are exceptional phytohormones, as they do not undergo long‐distance transport between plant organs. However, knowledge about the function of the enzymes that catalyse BR biosynthesis (particularly its early stages) in cereal crops remains limited. Therefore, this study identifies and analyses the function of the *HvDWARF5* (*HvDWF5*) gene, involved in the early stage of BR biosynthesis in barley (*Hordeum vulgare*), an important cereal crop, using the TILLING (Targeting Induced Local Lesions IN Genomes) approach. The detailed functional analysis allowed for the identification of various mutations in different gene fragments. The influence of these mutations on plant architecture, reproduction, and yield was characterised. Moreover, effects of the missense and intron retention mutations on sequence and splicing of the *HvDWF5* transcript, sequence and predicted structure of the encoded HvDWF5 enzyme, and accumulation of endogenous BR were determined. Some of the barley mutants identified in this study showed semi‐dwarfism, a trait of particular importance for cereal breeding and yield. However, unlike other BR mutants in cereals, this did not negatively affect grain size or weight. It indicated that mutations in this gene allow for a balance between plant height reduction and maintenance of grain size. Thus, the results of this study provide a novel insight into the role of the *HvDWF5* gene in the BR biosynthesis‐dependent regulation of architecture and reproduction of the important cereal crop – barley.

## 1 INTRODUCTION

Brassinosteroids (BRs) constitute a class of steroid phytohormones identified in the 1970s (Mitchell et al., 1970; Grove et al., 1979; Clouse, 2015). Initially, the influence of BRs on plant development was mainly associated with a positive effect on cell elongation. However, further studies indicated that BRs regulate a broad range of physiological and developmental processes in plants (Tong & Chu, 2018; Nolan et al., 2020). Noteworthy, BRs are exceptional in comparison with other phytohormones, as they do not undergo long‐distance transport between plant organs (Symons et al., 2008). However, recent studies have provided insights into BR export from cells and short‐distance (cell‐to‐cell) transport (Vukasinovic & Russinova, 2018; Wang et al., 2023; Ahmar & Gruszka, 2024; Ying et al., 2024). It was reported that BR biosynthesis takes place in all plant tissues and organs, but BR accumulation varies depending on species, tissue and development stage (Clouse & Sasse, 1998, Kanwar et al., 2017).

The process of BR biosynthesis has been extensively studied for the last two decades, mainly in the dicot model species ‐ *Arabidopsis thaliana* (Shimada et al., 2001; Fujioka & Yokota, 2003; Vriet et al., 2012; Chung & Choe, 2013; Bajguz et al., 2020). It is known that the BR biosynthesis constitutes a part of the broader biochemical process – biosynthesis of sterols (Bajguz et al., 2020). During sterol biosynthesis, the pathway splits into two branches: one leads to the production of sitosterol and stigmasterol, which are incorporated into cellular membranes, while the other results in BR synthesis (Lindsey et al., 2003; Bajguz et al., 2020). The BR biosynthesis is a complicated and multi‐step biochemical process catalysed by numerous enzymes. During this process, intermediates are gradually transformed into consecutive BR forms, which show increasing biological activity (Joo et al., 2012; Bajguz et al., 2020). However, our current knowledge about the BR biosynthesis and its regulation in other species, including cereal crops, is rather limited compared to Arabidopsis (Gruszka, 2019). Nevertheless, some of the BR biosynthesis genes have been functionally characterised in cereals, such as rice (*Oryza sativa*) (Hong et al., 2002; Hong et al., 2005; Sakamoto et al., 2006; Sakamoto et al., 2012; Li et al., 2018; Zhan et al., 2022), maize (*Zea mays*) (Tao et al., 2004; Liu et al., 2007; Hartwig et al., 2011; Makarevitch et al., 2012; Sun et al., 2021), and barley (*Hordeum vulgare*) (Gruszka et al., 2011; Dockter et al., 2014; Gruszka et al., 2016b).

Although insight into the several enzymatic steps operating in the downstream part of the BR biosynthesis has been obtained in the above‐mentioned cereal species, our current knowledge about the function of the enzymes which catalyse reactions at the early stages of the BR biosynthesis remains very limited, even in rice which is regarded as a model for cereals. One of these enzymes is DWARF5 (DWF5) which in Arabidopsis functions as 7‐dehydrocholesterol reductase catalysing the production of isofucosterol during the biosynthesis of sterols, which are incorporated into cellular membranes and regulating their physicochemical properties, as well as production of the BR precursor ‐ 24‐methylenecholesterol at the early stage of the BR biosynthesis (Choe et al., 2000; Lindsey et al., 2003; Bajguz et al., 2020). The activity of the 7‐dehydrocholesterol reductase was reported in the endoplasmic reticulum (Silvestro et al., 2013). Mutations in the *DWF5* gene, which led to defects of the encoded enzyme in Arabidopsis, resulted in changes in the plant architecture (dwarfism and semi‐dwarfism), as well as abnormalities in reproductive development, seed appearance, and germination (Choe et al., 2000). This indicates that proper function of the DWF5 enzyme is crucial for the regulation of plant stature, development, and reproduction. These features are of particular importance in crop species, as some of the semi‐dwarf BR mutants have already proven very valuable in cereal breeding programs. It has also been suggested that new semi‐dwarf cereal mutants defective in the BR biosynthesis or BR signalling may constitute an alternative in future breeding programs due to their erect stature, improved tolerance to environmental stresses, and enhanced nitrogen‐use efficiency (Chono et al., 2003; Saisho et al., 2004; Sakamoto et al., 2006; Dockter et al., 2014; Gruszka et al., 2020; Song et al., 2023).

Thus, the characterisation of the mechanisms of the BR biosynthesis‐dependent regulation of plant architecture, development, reproduction, and yield is crucial in cereals. Noteworthy, functional (mutational) analysis of a homologue of the *DWF5* gene has never been performed in any cereal species. Therefore, in this study, the identification and functional characterisation of the *HvDWF5* gene in barley was performed using the TILLING (Targeting Induced Local Lesions IN Genomes) approach. As a result of the study, various types of mutations located in different regions of the *HvDWF5* gene were identified. Effects of the identified alleles on the encoded transcript and enzyme variants, accumulation of BRs, and stature and reproduction of the mutants were characterised. Thus, results of this study provided a novel insight into the role of the *HvDWF5* gene in the BR biosynthesis‐dependent regulation of architecture and reproduction of barley as an important cereal species.

## 2 MATERIALS AND METHODS

### 2.1 Plant material

**FIGURE 1:**
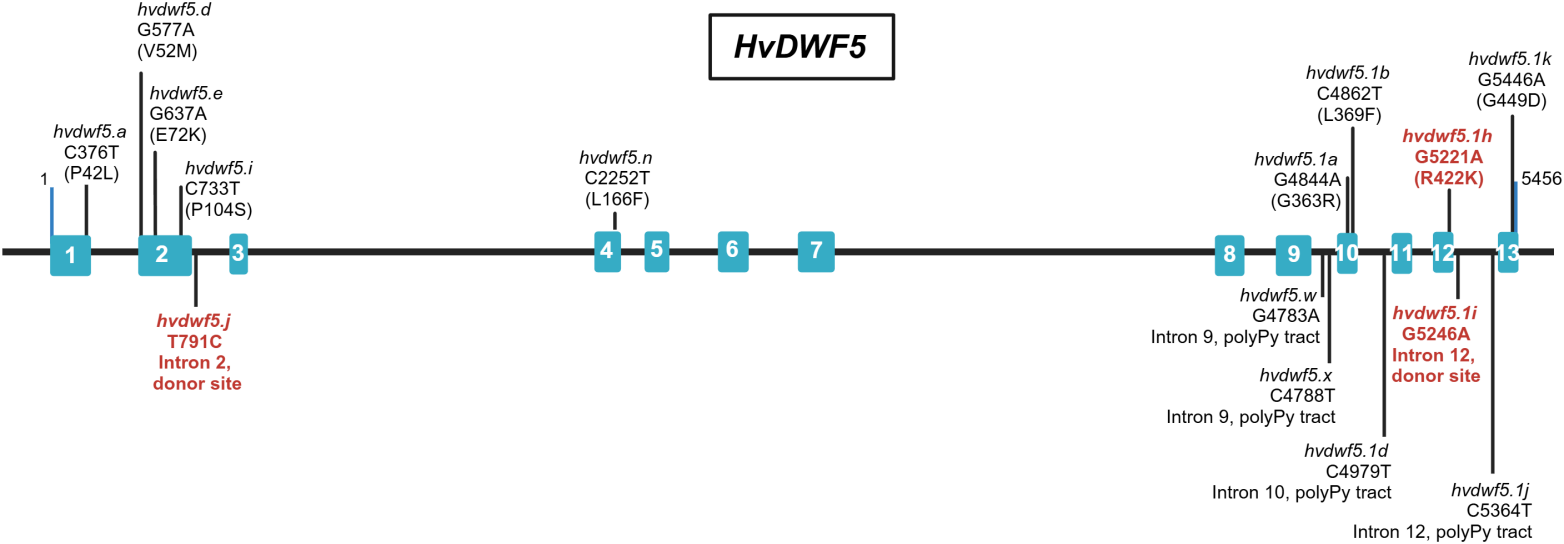
The *HvDWF5* gene structure with marked positions of the identified mutations within exons (blue rectangles) and introns (black horizontal lines). Mutations which result in the height reduction of the mutant plants are shown in red and bolded, and characterised in detail in this study.

Barley (*Hordeum vulgare*) mutants developed through the TILLING approach at the Department of Plant Genetics and Functional Genomics (University of Silesia, Poland) were used in this study. The mutants carry nucleotide substitutions within the coding regions (alleles *hvdwf5.a, hvdwf5.d, hvdwf5.e, hvdwf5.i, hvdwf5.n, hvdwf5.1a, hvdwf5.1b, hvdwf5.1 h, hvdwf5.1 k*) and non‐coding regions (alleles *hvdwf5.j, hvdwf5.w, hvdwf5.x hvdwf5.1d, hvdwf5.1i, hvdwf5.1j*) of the *HvDWF5* gene (Figure 1, Table 1). These mutants are included in the HorTILLUS population of barley. Our previous article extensively described the HorTILLUS mutant population, its development, and the TILLING method (Szurman‐Zubrzycka et al., 2018). In the analyses and experiments carried out, homozygous mutant lines were used for each of the identified alleles. To achieve homozygosity of these mutant lines, the experiments were performed on the homozygous mutant lines of the M_4_ or M_5_ generation. Moreover, in each generation, from M_3_ to M_5_, phenotypes of the homozygous mutants were monitored. In the case of each of the alleles and the homozygous mutant lines, the phenotypes remained stable from generation to generation (up to M_4_ or M_5_). For each of the analysed alleles (except *hvdwf5.1i*), several homozygous mutant lines were identified and included in the analyses and experiments of this study. The homozygous lines were kept separate from the M_2_ to the M_4_/M_5_ generations. Importantly, homozygous mutant lines representing specific allele exhibited the same phenotype. This confirms that the mutations analysed in this study are responsible for the observed mutant phenotypes. In the *hvdwf5.1i* allele, a single homozygous *hvdwf5.1i* mutant was identified.

**TABLE 1:** The identified mutations within the *HvDWF5* gene and their impact on the sequence of encoded protein or splicing process, as well as on the phenotype (height) of the mutant plants. Mutations which resulted in the plant height reduction are bolded and characterised in detail in this study.

Allele	Position of mutation	Effect of Mutation	Phenotype
*hvdwf5.a*	C376T (Exon 1)	P42L	Normal
*hvdwf5.d*	G577A (Exon 2)	V52M	Normal
*hvdwf5.e*	G637A (Exon 2)	E72K	Normal
*hvdwf5.i*	C733T (Exon 2)	P104S	Normal
***hvdwf5.j***	**T791C (Intron 2, donor site)**	**Alteration in splicing**	**Semi‐dwarf**
*hvdwf5.n*	C2252T (Exon 4)	L166F	Normal
*hvdwf5.w*	G4783A (Intron 9, polyPy tract)	‐	Normal
*hvdwf5.x*	C4788T (Intron 9, polyPy tract)	‐	Normal
*hvdwf5.1a*	G4844A (Exon 10)	G363R	Normal
*hvdwf5.1b*	C4862T (Exon 10)	L369F	Normal
*hvdwf5.1d*	C4979T (Intron 10, polyPy tract)	‐	Normal
***hvdwf5.1 h***	**G5221A (Exon 12)**	**R422K**	**Semi‐dwarf**
***hvdwf5.1i***	**G5246A (Intron 12, donor site)**	**Alteration in splicing**	**Dwarf**
*hvdwf5.1j*	C5364T (Intron 12, polyPy tract)	‐	Normal
*hvdwf5.1 k*	G5446A (Exon 13)	G449D	Normal

### 2.2 Verification of mutations of the *HvDWF5* gene in homozygous mutant plants

The homozygous mutant plants were grown in a greenhouse under a 16‐h photoperiod and 400 μmol m^−2^ s^−1^ light intensity under sodium lamps HPS Philips SON‐T AGRO 400 W. In order to confirm the occurrence of mutations within the *HvDWF5* gene, the DNA extractions followed by PCR amplifications were performed. Leaves of 3‐week‐old seedlings of the homozygous mutants and the ‘Sebastian’ cultivar (reference) served as a material for DNA isolation, carried out according to the REDExtract‐N‐Amp™ Plant PCR Kit (Sigma‐Aldrich) protocol. PCR primers were designed using the Jellyfish software (LabVelocity) based on the *HvDWF5* gene sequence (acc no. HORVU.MOREX.r3.3HG0320030) derived from the Ensembl Plants database [https://plants.ensembl.org/index.html]. The PCR primer sequences and PCR amplification profiles are available in Tables S1 and S2. The PCR products were sequenced (outsourced service provided by Genomed, Warsaw), and the sequences analysed using a licensed CodoneCode Aligner tool.

### 2.3 RNA extraction, reverse transcription and RT‐PCR

In order to determine the impact of mutations within introns of the *HvDWF5* gene on putative abnormalities in the transcript splicing process, RT‐PCR were preformed, preceded by RNA extraction, purification and reverse transcription. Leaves and roots of 8‐day‐old seedlings of the homozygous mutants and the ‘Sebastian’ cultivar (reference) were grown in aeroponic conditions and served as a material for RNA isolation performed according to the TRIpure Reagent protocol (Total RNA Extraction Kit, Roche). The concentration and purity of the isolated RNA were measured using Spectrophotometer ND‐1000 (NanoDrop).

The obtained RNA isolates were subjected to reverse transcription reaction according to the protocol of the NG dART RT kit (EURx, Poland), preceded by RNase‐free DNase (Promega) treatment. The RT‐PCR experiments were based on cDNA derived from the reverse transcription. The sequences of primers applied during the RT‐PCR experiments and the RT‐PCR profiles are available in Tables S1 and S3.

Information about the *HvDWF5* transcript variants was retrieved from the Ensembl Plants (Yates et al., 2022, https://plants.ensembl.org/index.html) and EoRNA (Milne et al., 2021; https://ics.hutton.ac.uk/eorna/index.html) databases. To specify the abundance of particular transcripts in analysed tissues, only experiments in which plants were not subjected to any stress conditions were considered.

### 2.4 Measurement of endogenous brassinolide concentration

For quantification of the endogenous brassinolide (BL) content in the analysed genotypes, fragments of leaf tissues were collected from the second (developmentally) leaves when plants of each genotype developed two or three tillers (at the same developmental stage). In each genotype, the leaf fragments were collected from the first (developmentally) tiller. Each sample represented 3 leaf fragments collected from 3 plants of a given genotype (pooled together) per 1 biological replicate. One biological replicate represented a different homozygous line developed from the M_2_ generation. In each genotype, the leaf sampling was performed in 3 biological replicates. Therefore, in each genotype, the leaf fragments were collected from 9 plants in total. Plants of the analyzed genotype from which the leaf fragments were collected were grown in a greenhouse under a 16‐h photoperiod and light conditions mentioned above. Leaf tissues (1 g F.W. per sample) were homogenized in 20 mL 80% methanol (MeOH) for 12 h at 4°C followed by centrifugation (36 670 *g*, 10 min, 4°C). The obtained supernatants were supplemented with an internal standard mixture containing 10 pmol each of ^2^H_3_‐labelled BRs and loaded on the Discovery DPA‐6S columns (Supelco, Bellefonte, PA 16823, USA) as described in previous articles (Gruszka et al., 2016a; Tarkowská et al., 2016). The filtered volumes were evaporated to dryness *in vacuo*. The obtained residues were dissolved in 75 μL of 100% MeOH by vortexing, followed by sonication for 5 min, and made up to 1 mL with PBS buffer (50 mM NaH_2_PO_4_ and 15 mM NaCl, pH 7.2). The samples were loaded on columns containing antibodies against BRs (IAC columns) and eluted using 3 mL of ice‐cold MeOH (−20°C). The elution fraction was evaporated to dryness in a benchtop concentrator (CentriVap R Acid‐Resistant benchtop concentrator, Labconco Corp.), and the analysis was performed with the use of a Ultra High Performance Liquid Chromatography UHPLC–MS/MS (Tarkowská et al., 2016). Each measurement was performed in three repetitions.

### 2.5 *In silico* analysis of the impact of identified mutations on the functionality of the encoded protein

Conservation of amino acid residues of the DWF5 proteins was determined based on the alignment of sequences of 9 orthologs from the following dicot and monocot species: *Aegilops tauschii*, *Arabidopsis thaliana, Brachypodium distachyon*, *Helianthus annuus*, *Hordeum vulgare*, *Oryza sativa*, *Sorghum bicolor*, *Triticum aestivum*, and *Zea mays* retrieved from the NCBI (https://www.ncbi.nlm.nih.gov/) and TAIR (https://www.arabidopsis.org/) databases. The DWF5 protein sequences were aligned with the Clustal Omega tool (https://www.ebi.ac.uk/Tools/msa/clustalo/; Sievers et al., 2011). The sequences of *HvDWF5* transcripts with incorporated introns as a result of the identified mutations within donor sites were translated *in silico* using Jellyfish software (LabVelocity). The impact of identified mutations on the activity of conserved functional domains was analysed using the ScanProsite (https://prosite.expasy.org/scanprosite/; De Castro et al., 2006) and InterPro (https://www.ebi.ac.uk/interpro/; Paysan‐Lafosse et al., 2023) tools. The distribution of transmembrane domains was predicted using the DeepTMHMM 1.0.24 (https://dtu.biolib.com/DeepTMHMM; Hallgren et al., 2022) program. The 3D structures of the analysed versions of the HvDWF5 protein were analysed using I‐TASSER (https://zhanggroup.org/I-TASSER/; Yang & Zhang, 2015), based on the selection of structures with the highest C‐score parameter. The I‐TASSER tool was also used to identify amino‐acid residues within the HvDWF5 protein that take part in binding ligands. The 3D structures were visualized and labelled using the UCSF Chimera (https://www.cgl.ucsf.edu/chimera/; Pettersen et al., 2004) program. The secondary structures were predicted using the PSIPRED tool (http://bioinf.cs.ucl.ac.uk/psipred/; Buchan & Jones, 2020) and the impact of identified amino acid substitution was verified with the use of I‐Mutant2.0 (https://folding.biofold.org/i-mutant/) tool.

### 2.6 Data visualisation

Plots concerning the phenotypic characterization of the *hvdwf5.1i, hvdwf5.j* and *hvdwf5.1 h* mutant plants, as well as the endogenous contents of brassinolide (BL) were created with the use of the R 4.4.0 software and the ggstatsplot package (Patil, 2021). The rest of the plots were prepared using the ggplot2 and gridExtra packages (Wickham, 2016; Auguie & Antonov, 2022).

### 2.7 Statistical analysis

All statistical calculations were carried out in STATISTICA version 12.0 (StatSoft Inc. 2014) or PAST 4.03. When the dataset had a normal distribution, a *t*‐test was conducted to compare each of the analysed mutant plants with the reference, with *p* < 0.05 (*) and *p* < 0.01 (**) considered to be significantly different. Otherwise, when the dataset did not meet the requirement of normal distribution for the *t*‐test, a non‐parametric Mann–Whitney U test was performed (*p* < 0.05 (*) and *p* < 0.01 (**)). During the analysis of morphology and weight of grains, the grain length, width and thickness parameters were calculated based on 50 grains (*n* = 50), and the 100‐grain weight parameter was determined based on 10 replicates (*n* = 10).

## 3 RESULTS

### 3.1 Phenotypic characterization of the hvdwf5 mutants

**FIGURE 2:**
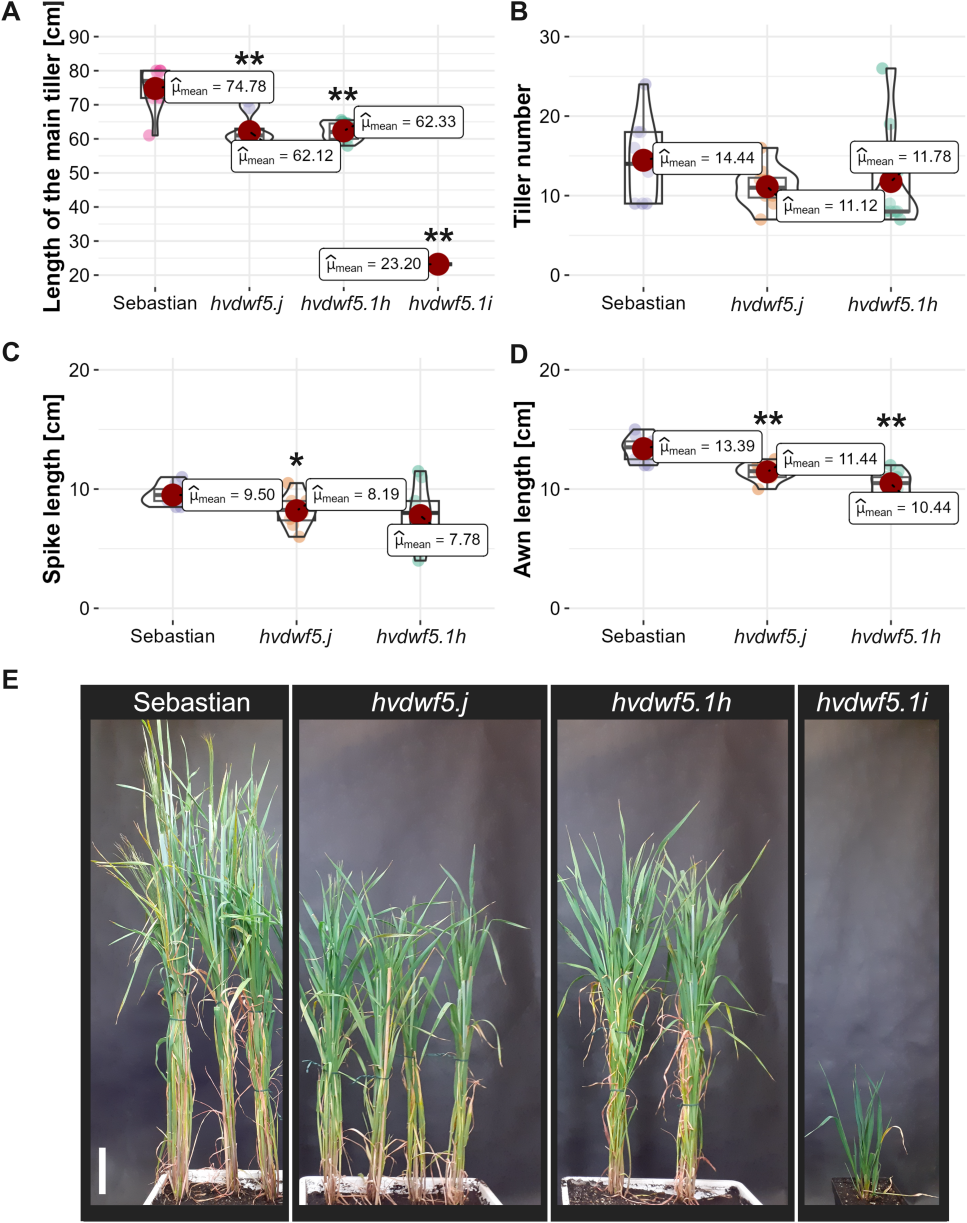
Phenotypic characterisation of the *hvdwf5.j*, *hvdwf5.1h*, and *hvdwf5.1i* mutant plants compared with the reference cultivar ‘Sebastian’. The measurements were performed on 10 plants of each genotype (except the *hvdwf5.1i* mutant) at the full maturity of plants (at harvest). Spike‐producing tillers were taken into account when determining the tiller number, therefore, the *hvdwf5.1i* mutant (sterile) is not represented in panels B, C, and D. (E) Comparison of plant stature between the analysed genotypes (9‐week‐old plants). Scale bar = 10 cm. Asterisks represent level of significance (** indicates *p* < 0.01; * indicates *p* < 0.05).

A series of alleles in the barley *HvDWF5* gene (HORVU.MOREX.r3.3HG0320030) was identified using the TILLING approach. Fifteen homozygous mutant lines, harbouring either missense mutations or mutations located in the introns, were developed and analysed in terms of the plant height (Figure 1, Table 1), as (semi‐)dwarfism is the primary feature which correlates with disturbance in the BR homeostasis (including BR deficiency). Among the identified alleles, several carried the nucleotide substitutions resulting in missense mutations located in the 1st (allele *hvdwf5.a*), 2nd (alleles *hvdwf5.d*, *hvdwf5.e*, *hvdwf5.i*), 4th (allele *hvdwf5.n*), 10th (alleles *hvdwf5.1a* and *hvdwf5.1b*), 12th (allele *hvdwf5.1h*) and 13th (allele *hvdwf5.1k*) exon of the *HvDWF5* gene (Figure 1, Table 1). As far as the mutations in the non‐coding sequence are concerned, two were located in the donor sites of the 2nd (allele *hvdwf5.j*) and 12th (allele *hvdwf5.1i*) intron and four putatively located in polypyrimidine tracts (PPTs) of the intron 9th (alleles *hvdwf5.w* and *hvdwf5.x*), intron 10th (allele *hvdwf5.1d*), and intron 12th (allele *hvdwf5.1j*). Given the fact that three mutations (alleles *hvdwf5.j*, *hvdwf5.1h* and *hvdwf5.1i*) resulted in the main tiller length reduction and thus the height reduction of mutant plants (Figure 2A, E, Table 1), which is the critical feature regarding BR‐mutants, these mutations were taken into account for further analysis. The more detailed phenotypic characterisation of the homozygous mutants carrying these alleles encompassed analysis of the following traits: the length of the main tiller, tiller number, spike length and awn length. Some mutations did not have a statistically significant impact on plant phenotype (Table 1), and their phenotypes are shown in Figure S1.

The *hvdwf5.j* and *hvdwf5.1h* mutants displayed the semi‐dwarf phenotype with statistically significant (*p* < 0.01) height reduction by 17% on average when compared with plants of the ‘Sebastian’ cultivar (Figure 2A). Interestingly, among the progeny of heterozygous M_2_ plants, which comprised of 112 individuals, only one homozygous *hvdwf5.1i* mutant was identified. This mutant exhibited a severely dwarfed phenotype, reaching only 31% of the height of the reference cultivar (Figure 2A, E) and was completely sterile. Noteworthy, previous reports indicated the crucial involvement of BR signalling and biosynthesis in vascular bundles development (Choe et al., 1999; Hong et al., 2002; Ibanes et al., 2009; Lee et al., 2019; Oh et al., 2020). As the *hvdwf5.1i* mutant displayed the most severe dwarf phenotype compared to the other mutants identified in this study, the vascular bundles organisation was analysed in the *hvdwf5.1i* mutant. The *hvdwf5.1i* mutant displayed an altered organisation of these tissues, particularly the increased number of vascular bundles was observed (Figure S2), which is consistent with the previous study (Wu et al., 2016).

The *hvdwf5.j* and *hvdwf5.1h* mutants did not show any significant alteration in tiller number (Figure 2B). It was observed that the *hvdwf5.j* mutant developed shorter (*p* < 0.05) spikes (Figure 2C). The *hvdwf5.j* and *hvdwf5.1h* mutants were characterised by the significantly (*p* < 0.01) reduced awn length (Figure 2D), but more importantly these mutants were fully fertile.

**FIGURE 3:**
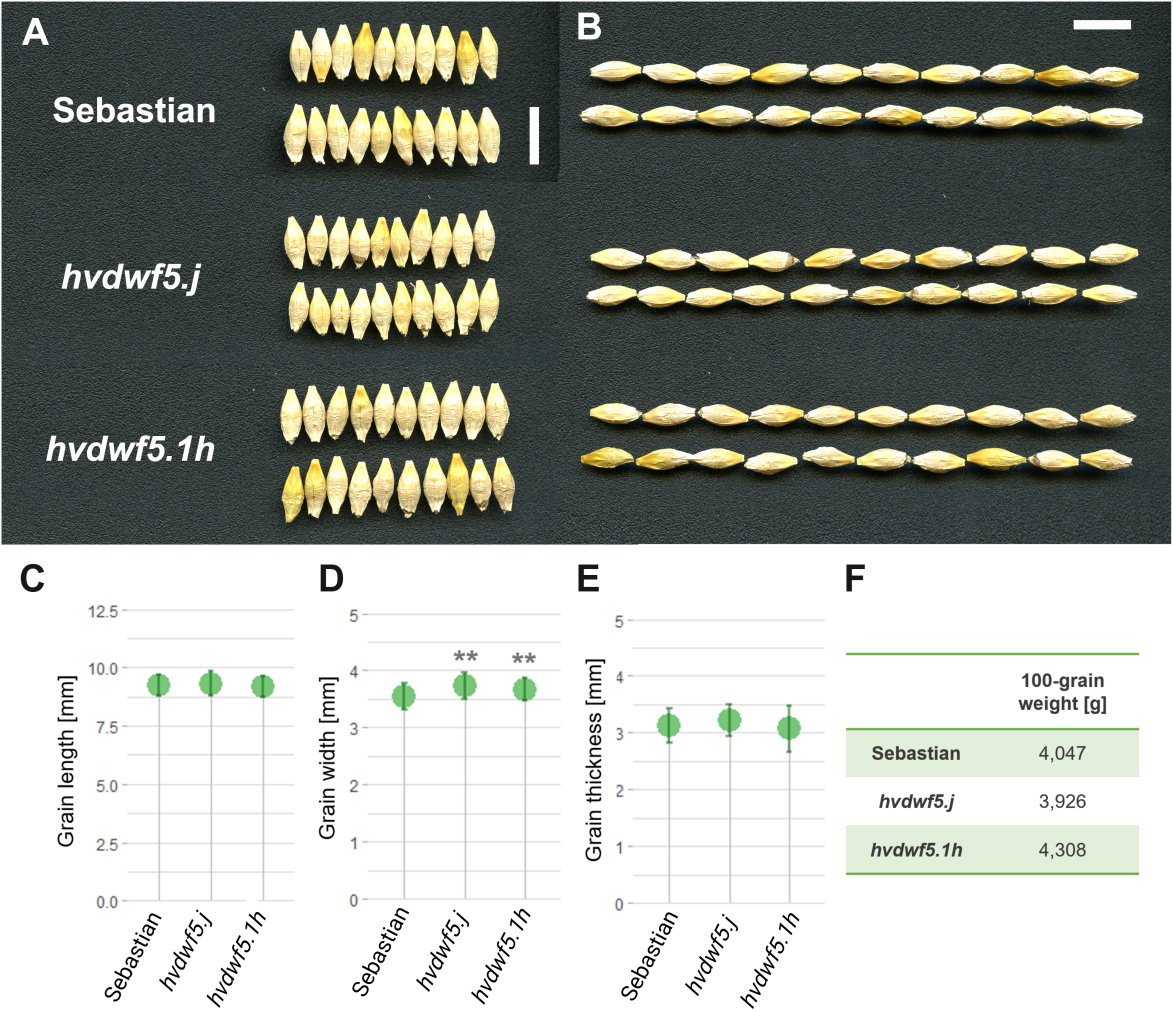
Characteristics of grains produced by the analysed mutants and the reference cultivar ‘Sebastian’. (A‐B) Morphology of grains produced by the *hvdwf5.j* and *hvdwf5.1h* mutants and the reference ‘Sebastian’. Scale bar = 1 cm. Due to complete sterility of the *hvdwf5.1i* mutant, this genotype is not represented in this figure. (C‐F) Grain length, width, thickness, and 100‐grain weight in the *hvdwf5.j* and *hvdwf5.1h* mutants and the reference ‘Sebastian’. The mean values are presented for each genotype, with error bars representing standard deviation. The grain length, width, and thickness parameters were calculated in each genotype on the basis of 50 grains (*n* = 50) derived from 10 plants of this genotype, whereas 100‐grain weight was determined in each genotype on the basis of measurement of 10 batches of 10 grains, where 1 batch represents 1 plant of a given genotype; 10 replicates (*n* = 10). Asterisks represent level of significance (** indicates p < 0.01).

An accumulating body of evidence indicates that BRs have a pivotal role in the regulation of various agronomic traits, including grain size (Tong & Chu, 2018). However, most of the cereal BR mutants characterised by defects in the function of enzymes catalysing the BR biosynthesis or acting as the positive regulators of the BR signalling, produced smaller grains (Hong et al., 2005; Guo et al., 2014; Yuan et al., 2017; Zhang et al., 2021; Xu et al., 2022; Sun et al., 2023). Interestingly, the mutant with the *hvdwf5.1h* allele displayed slightly heavier grains, however, the difference was not significant. The grain length and thickness were not affected, but the grains of the *hvdwf5.j* and *hvdwf5.1h* mutants were wider (Figure 3A‐E). Importantly, the 100‐grain weight of these mutants was similar to the reference cultivar (Figure 3F). These results indicate that the identified alleles cause some of the typical BR‐deficient or BR‐insensitive mutant phenotypes in barley, i.e. decrease in plant height and shortened awns (Dockter et al., 2014). However, at the same time, the negative effect of the BR deficiency on grain size (reported in the above‐mentioned cereal BR mutants) was not observed in the *hvdwf5.j* and *hvdwf5.1h* mutants. Therefore, the results of this study provided a promising material for further genetic manipulations aimed at the improvement of cereal crops, particularly through achieving a balance between semi‐dwarfism and maintenance of grain size and weight in barley.

### 3.2 Molecular characterization of the *hvdwf5* mutants harbouring mutations within donor sites of the introns

**FIGURE 4:**
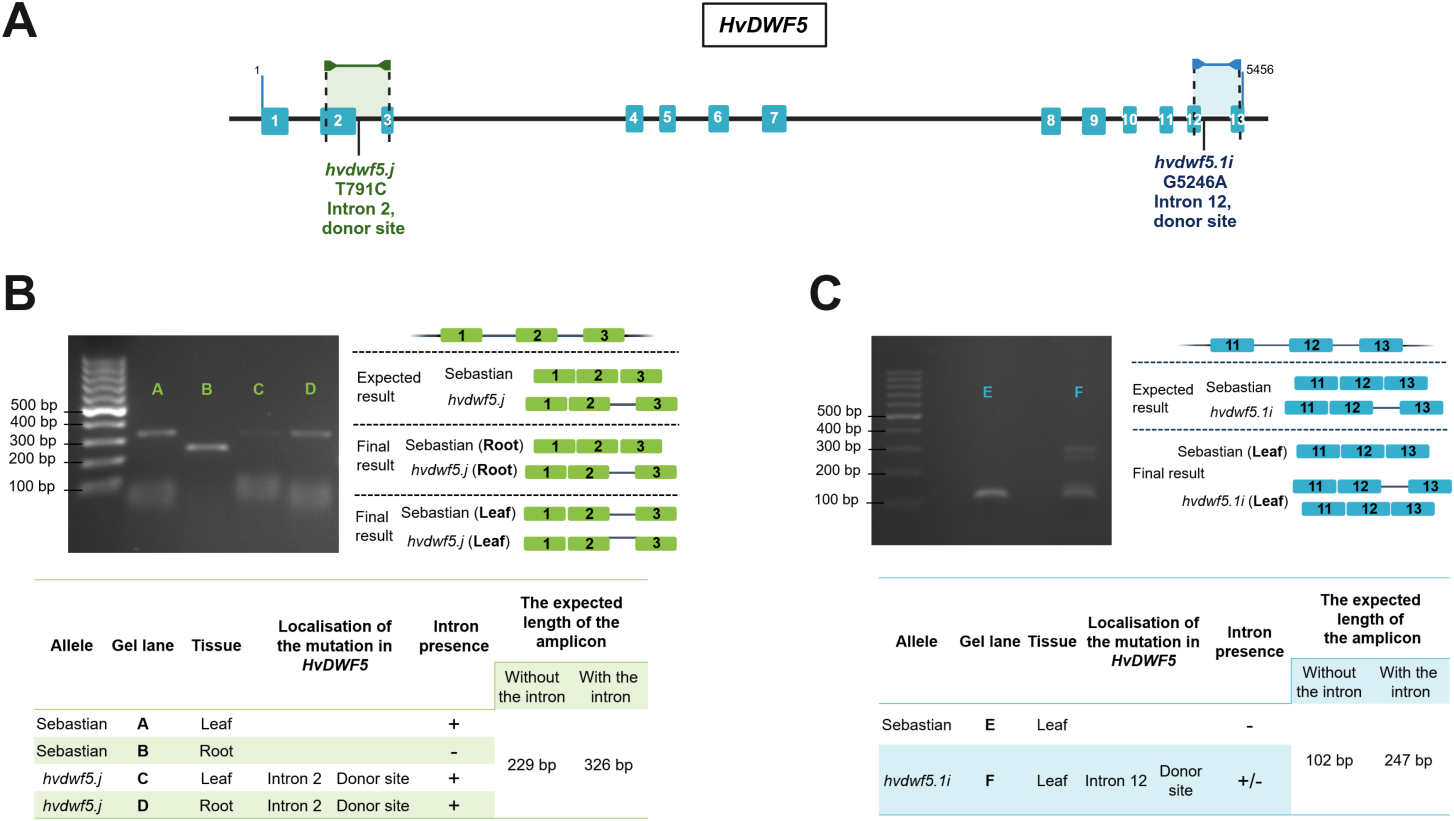
Reverse transcription‐polymerase chain reaction (RT‐PCR) analysis of the 2nd and 12th intron retention within the *HvDWF5* transcripts produced by the *hvdwf5.j* and *hvdwf5.1i* mutants, respectively. (A) The *HvDWF5* gene structure with marked positions of the identified mutations within donor sites of the 2nd and 12th introns, above which the position of primers which were used for amplification of the transcript regions containing these introns are indicated by arrows. (B‐C) The results of RT‐PCR reactions on the basis of cDNA derived from roots and leaves of 8‐day‐old seedlings of the *hvdwf5.j* mutant and leaves of the *hvdwf5.1i* mutant, and from the respective organs of the reference cultivar ‘Sebastian’.

To gain an insight into the molecular mechanism underlying the phenotype of mutants carrying substitutions in the donor sites of the 2nd intron (T791C, allele *hvdwf5.j*) and the 12th intron (G5246A, allele *hvdwf5.1i*), the potential retention of these introns within transcripts extracted from roots and leaves of 8‐day‐old seedlings of the *hvdwf5.j* mutant and leaves of the *hvdwf5.1i* mutant was investigated using the Reverse Transcription‐Polymerase Chain Reaction (RT‐PCR) approach (Figure 4A). The results of this experiment showed that the splicing process is indeed affected in these mutants. The expected length of the amplicon when the 2nd intron is properly spliced amounts to 229 bp, whereas the amplicon with incorporated 2nd intron was expected to be 326 bp long. Expression profile analysis and amplicon sequencing indicated that the *HvDWF5* transcript in the *hvdwf5.j* mutant contains the 2nd intron retained in both of the analysed tissues (Figure 4B). Surprisingly, the retention of the 2nd intron was also observed in the *HvDWF5* transcript extracted from leaves of the ‘Sebastian’ cultivar (reference). This result suggested an organ‐specific preference for the *HvDWF5* transcript variants during the alternative splicing in this barley cultivar. In the case of the *hvdwf5.1i* mutation, apart from the amplicon of similar length (102 bp) as in the reference, an additional 247 bp‐long amplicon was obtained, which is of the expected length of the amplicon containing the 12th intron retained within the *HvDWF5* transcript (Figure 4C). Taken together, these results indicated that both *hvdwf5.j* and *hvdwf5.1i* mutations have a significant impact on the splicing process (intron retention) of the *HvDWF5* transcript.

**FIGURE 5:**
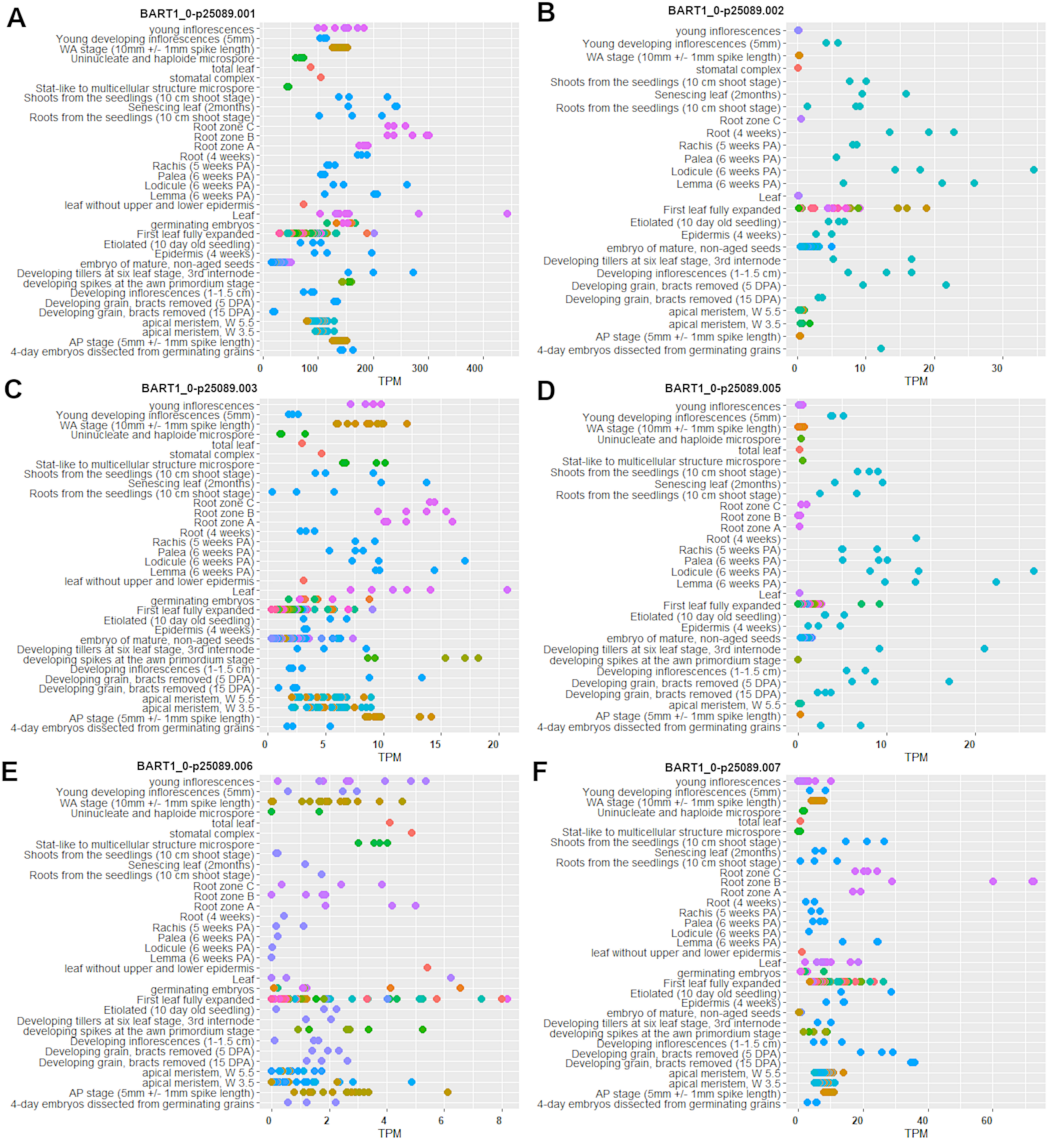
Expression profile analysis of the selected *HvDWF5* transcript variants (TPM ‐ transcript per million). The expression data for these transcripts in various tissues were retrieved from the EoRNA database.

The retention of the 2nd intron also in the reference genotype (‘Sebastian’) and the occurrence of additional amplicon products in leaves of the *hvdwf5.1i* mutant (Figure 4C) raised our curiosity about alternatively spliced transcripts of *HvDWF5*. Thus, to verify the hypothesis, the information about transcript variants of *HvDWF5* was retrieved from the Ensembl Plants (Yates et al., 2022, https://plants.ensembl.org/index.html) and EoRNA (Milne et al., 2021; https://ics.hutton.ac.uk/eorna/index.html) databases. Interestingly, based on data retrieved from the Ensembl Plants database, the *HvDWF5* gene (HORVU.MOREX.r3.3HG0320030) has 4 splice variants, while in the EoRNA database, 13 splice variants of the *HvDWF5* gene have been reported. Furthermore, the expression level of these transcripts in various tissues was also analyzed with the use of the EoRNA database. Each splice variant is characterised by different expression level and BART1_0‐p25089.001, which is also the longest (3543 bp), stands out in terms of expression level, particularly in leaves and roots (Figure 5A). Additionally, some of the transcripts are also expressed in lodicule and lemma (BART1_0‐p25089.001, BART1_0‐p25089.002, BART1_0‐p25089.003, BART1_0‐p25089.005) (Figure 5A‐D). Expression profiles of all the *HvDWF5* transcript variants are shown in Figure S3. Therefore, this analysis indicated that the *HvDWF5* gene has several splice variants, and the expression of particular transcripts is organ‐specific (confirmed by the RT‐PCR results in this study). These results may explain the presence of additional amplicon products obtained in the RT‐PCR reaction, however, further research is needed to clarify the underlying mechanisms fully.

### 3.3 Analysis of endogenous BR concentration

**FIGURE 6:**
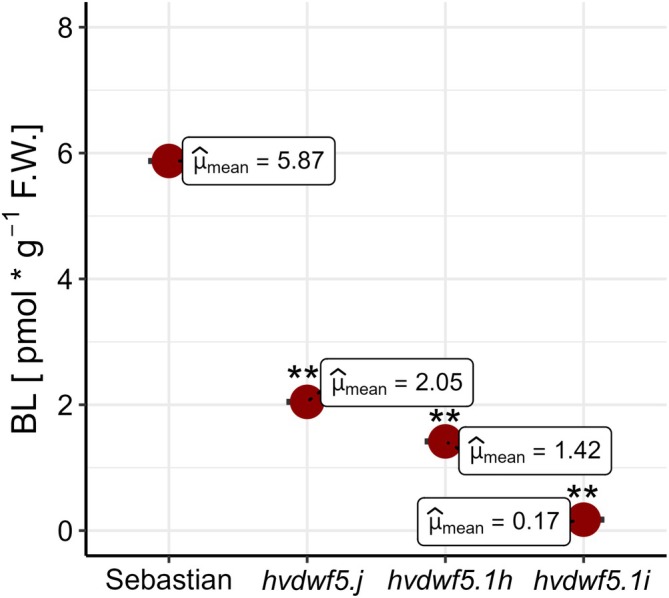
The endogenous contents of brassinolide (BL) in the analyzed genotypes. The mean values of three replicates of each measurement are presented for each genotype. Asterisks represent level of significance (** indicates p < 0.01).

Based on the above‐mentioned reports that DWF5 in Arabidopsis is involved in the early stage of the BR biosynthesis (Choe et al., 2000; Lindsey et al., 2003; Bajguz et al., 2020), we assumed that the homologous HvDWF5 in barley may play similar role during the BR biosynthesis process. To verify this hypothesis, the impact of the *hvdwf5.j*, *hvdwf5.1i* and *hvdwf5.1h* mutations on the accumulation of the most biologically active form of BRs ‐ brassinolide (BL) was investigated, as abnormalities in the accumulation of biologically active BRs have a profound effect on plant phenotype. A similar approach was followed in another study on BR‐deficient barley mutants defective at various steps of the BR biosynthesis process (Dockter et al., 2014). In comparison with ‘Sebastian’, all analysed mutants exhibited significantly (*p* < 0.01) lower content of BL (Figure 6). The lowest accumulation of BL was observed in the *hvdwf5.1i* mutant, which was in accordance with the severity of the mutants' phenotypes which were described above. The obtained results validated the influence of identified mutations on the function of the HvDWF5 protein. This way, the importance of HvDWF5 in the BR biosynthesis pathway was confirmed.

### 3.4 Bioinformatics analysis of the impact of the identified mutations on the functionality of the encoded protein

**FIGURE 7:**
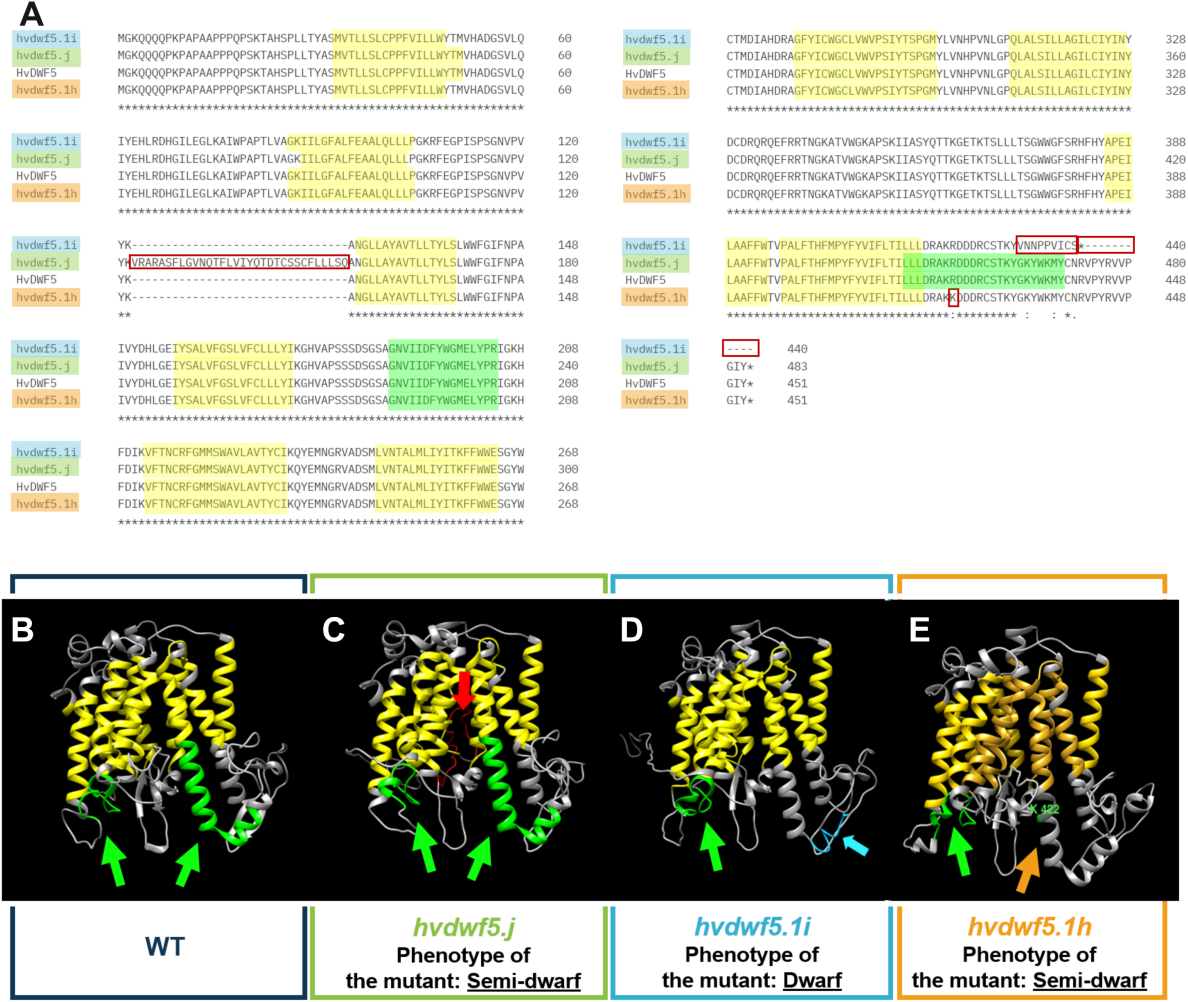
*In silico* analyses of the impact of the identified mutations on the sequence, structure, and activity of the encoded versions of HvDWF5. (A) Alignment of the HvDWF5 sequence and the versions of this protein encoded by the *hvdwf5.1i*, *hvdwf5.j* and *hvdwf5.1h* alleles. The alterations in amino acid sequence which resulted from the mutations are indicated by red frames. (B‐E) Visualisation of the 3D structures of HvDWF5 and the mutated versions of this protein, predicted based on the I‐TASSER and Chimera tools. The presence of two conserved sterol reductase functional domains of HvDWF5 (based on the ScanProsite and InterPro tools) and transmembrane domains (based on the DeepTMHMM tool) are indicated by green and yellow colours, respectively.

To characterise the impact of identified mutations on functionality of the HvDWF5 protein, several bioinformatics analyses were performed. As demonstrated above, the *hvdwf5.j* and *hvdwf5.1i* mutations resulted in the retention of the 2nd and 12th intron, respectively. Therefore, transcripts containing the incorporated intron sequences were also translated using Jellyfish. Intriguingly, the retention of the 2nd intron as a consequence of the *hvdwf5.j* mutation caused an insertion of the sequence encoding 32 amino acids, however, it did not change the codon reading frame (Figure 7A). On the contrary, *hvdwf5.1i* was found to be a frameshift mutation, and consequently, nine consecutive amino acids encoded in the mutated transcript were substituted and additionally premature stop codon was introduced into the coding sequence at the adjacent position (Figure 7A). Furthermore, using ScanProsite (https://prosite.expasy.org/scanprosite/) and InterPro (https://www.ebi.ac.uk/interpro/) tools the impact of *hvdwf5.j* and *hvdwf5.1i* mutations on the presence of two functional (sterol reductase) domains of HvDWF5 was predicted. Interestingly, the analysis revealed that the insertion of the 2nd intron (the *hvdwf5.j* allele) presumably affected the function of neither of these functional domains, nevertheless, their positions were altered (Figure 7C, Table 2) when compared with the wild‐type version of the HvDWF5 protein (Figure 7B, Table 2). Moreover, an altered distribution (localisation) of transmembrane domains was observed in the HvDWF5 protein version encoded by the *hvdwf5.j* allele (Table S4). On the contrary, the frameshift *hvdwf5.1i* mutation partially changed the sequence within the second sterol reductase domain (the above‐mentioned substitution of nine amino acids) and shortened the length of the encoded protein (Figure 7A). These results, along with the bioinformatics (ScanProsite and InterPro) analyses confirmed that this mutation with high probability influences the function of one of the two functional domains (Figure 7D, Table 2), what might constitute a cause of the observed severe phenotype of the *hvdwf5.1i* mutant. Additionally, both analysed mutations affected the secondary structure of the encoded versions of HvDWF5 (Figure S4).

**TABLE 2:** Impact of the identified mutations on the activity of two highly conserved functional domains of the HvDWF5 protein, predicted with the use of ScanProsite and InterPro tools.

	WT	*hvdwf5.j*	*hvdwf5.1i*	*hvdwf5.1 h*
Sterol reductase (I)	189–204	221–236	189–204	189–204
Sterol reductase (II)	415–438	447–470	‐	‐

**FIGURE 8:**
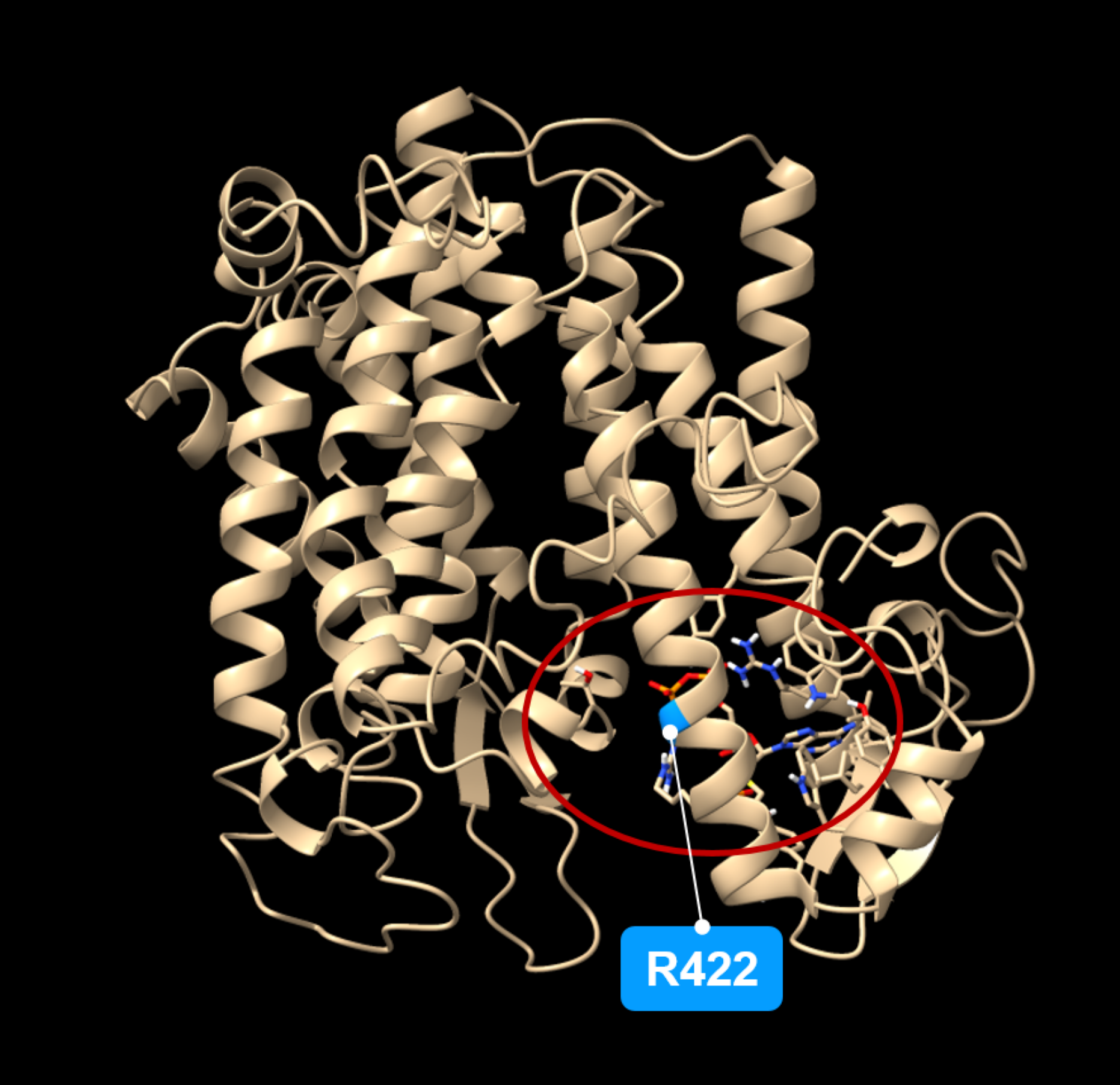
Visualisation of the 3D structure of HvDWF5 with the localisation of NADP binding site (indicated by red oval) predicted based on the I‐TASSER and Chimera tools. The arginine (R422) replaced in the *hvdwf5.1h* mutant is highlighted in blue.

As far as the consequences of the *hvdwf5.1h* mutation are concerned, the multiple sequence alignment unveiled that the replaced arginine (R422) is highly conserved across analysed monocot and dicot plant species (Figure S5). Using the I‐Mutant2.0 tool (https://folding.biofold.org/i-mutant/), it was also predicted that the R422K substitution decreased the stability of the encoded protein (value −0.92). Interestingly, the R422 residue is located within the second sterol reductase domain (Figure 7A). In a further analysis with the use of ScanProsite and Interpro tools, it was indicated that the R422K mutation may disrupt the functionality of the second sterol reductase functional domain (Table 2). These analyses validated the crucial importance of the R422 residue for the activity of this domain (Figure 7E). To get deeper insight into the role of R422 residue in the function of the HvDWF5 enzyme, the HvDWF5 protein sequences from the ‘Sebastian’ cultivar and the *hvdwf5.1h* mutant were analysed with the I‐TASSER programme. The analysis identified amino‐acid residues (ligand binding sites) that bind at least 5 different ligands (Table S5). Importantly, the R422 residue is located in the direct vicinity of one of the residues (D423), which are responsible for binding the nicotinamide adenine dinucleotide phosphate (NADP) molecule (Figure 8, Table S5). Additionally, the R422 residue is one of the residues binding a ligand which has not yet been characterized in detail (according to the I‐TASSER programme; Table S5). This indicated that the R422 residue is located at the position important for binding two ligands by the HvDWF5 enzyme.

Taken together, these results provided an insight into the impact of the identified mutations on the sequence, structure and functionality of the encoded protein. They indicated that each of these mutations influenced the HvDWF5 protein differently. The diverse impact of these mutations on the function of the HvDWF5 protein was confirmed by the decrease in the BL accumulation in the mutants (Figure 6) and their altered phenotypes (Figure 2, Table 1).

## 4 DISCUSSION

In this study, various types of mutations localised in different fragments of the *HvDWF5* gene were identified. These mutations also have diverse effects on the sequence of the encoded transcript and protein versions (Table 1). The first level of verification of the effects of these mutations was the phenotypic characterization of the homozygous mutants which carry each of these alleles. In the homozygous mutant plants with the *hvdwf5.1h* (R422K) allele, the length of the main tiller and length of awns were significantly (*p* < 0.01) lower than in the reference cultivar ‘Sebastian’ (Figure 2). Height of the *hvdwf5.1h* mutant plants reach 83% of the ‘Sebastian’ plant height. All seven *hvdwf5.1h* homozygous mutant plants showed the semi‐dwarf phenotype. Noteworthy, the (semi‐)dwarf plant stature and short awns are regarded as aspects of a unique combination of visible characters of the BR‐deficient and BR‐insensitive mutants of barley – the ‘ideotype’ of the BR mutants of barley (Dockter et al., 2014). Dwarfism or semi‐dwarfism (ranging from 22 to 53% of the height of wild‐type plants) was also observed in six *dwf5* mutants of Arabidopsis, which carried various loss‐of‐function mutations (frameshift mutation, perturbations of transcript splicing, nonsense mutations and missense mutation). Importantly, the *dwf5* mutants of Arabidopsis also showed reduction in the lengths of petioles, pedicels, and siliques. Moreover, the length of siliques was correlated with fertility, and longer siliques contained more seeds than shorter ones (Choe et al., 2000). Interestingly, changes in agronomic traits, such as tillering (Mori et al., 2002; Zhou et al., 2017; Huang et al., 2022) or size of inflorescence (Dockter et al., 2014; Zhou et al., 2017; Zhang et al., 2019) were previously observed in the BR biosynthesis mutants of cereals. However, in our study, the *hvdwf5.1h* mutant did not show any significant alteration in these parameters when compared with the ‘Sebastian’ cultivar (Figure 2). Importantly, the amino‐acid residue (R422) substituted in the *hvdwf5.1h* mutant is fully conserved among homologous sequences from monocot and dicot species (Figure S5), and also among the sterol reductases derived from such diverse species as human (*Homo sapiens*), rat (*Rattus norvegicus*), and yeast (*Saccharomyces cerevisiae*) (Choe et al., 2000). Moreover, our *in silico* analyses indicated that the mutation presumably significantly decreases the stability of the encoded enzyme and introduces some alterations in the arrangement of its secondary structural domains. Further analyses with two programs (ScanProsite and InterPro) indicated that in the enzyme version encoded by the *hvdwf5.1h* allele, the activity of the second sterol reductase functional domain is predicted to be disturbed. Noteworthy, the substituted R422 residue is located within this second functional domain (Figure 7A, E and Table 2). Thus, we postulated that such a significant alteration in the functional domain of the enzyme may influence the endogenous BR accumulation and, consequently, the semi‐dwarf phenotype of the *hvdwf5.1h* mutant. Indeed, the brassinolide (the active form of BR) accumulation was found to be significantly (several folds) decreased in the *hvdwf5.1h* mutant when compared with the reference cultivar ‘Sebastian’ (Figure 6). It indicated that the R422 residue is crucial for the activity of the second sterol reductase functional domain. As mentioned above, the analyses performed in this study indicated that the R422 residue is located at the adjacent position to one of the residues (D423) responsible for binding the NADP molecule (Figure 8, Table S5). Importantly, it was reported for DWF5 from Arabidopsis that the sterol reductase activity of this enzyme is NADPH‐dependent (Lecain et al., 1996). Additionally, R422 is one of the residues binding a ligand which has not yet been characterized in detail (Table S5). This indicated that the R422 residue is located at the position important for binding two ligands by the HvDWF5 enzyme. It was also reported in Arabidopsis that R403 (located at the corresponding position to R422 in barley) is located within a mixed charge cluster, which is conserved among various species (Choe et al., 2000). Noteworthy, although the *hvdwf5.1h* allele resulted in the semi‐dwarf phenotype, it did not negatively affect plant fertility. It is an important observation, as several previously described cereal mutants defective in the BR biosynthesis (due to loss‐of‐function mutation or gene downregulation) showed a reduction in plant fertility (Hong et al., 2005; Makarevitch et al., 2012; Wu et al., 2016; Zhou et al., 2017; Xu et al., 2022). Correlations between developmental defects of the *dwf5* mutants and the negative impact of mutations on the activity of the sterol reductase domains were also observed in Arabidopsis (Choe et al., 2000). However, the missense mutation identified in our study in the *hvdwf5.1h* allele has a much less severe effect on the mutant phenotype than the mutations identified in Arabidopsis (particularly the *dwf5‐2* and *dwf5‐3* alleles, which resulted in truncation of the encoded enzyme). The various types of mutations may explain the difference in the severity of the mutant phenotypes. Therefore, the results of our study indicated that the missense mutation in *HvDWF5* encoding the enzyme catalysing the early step of the BR biosynthesis may allow for the development of semi‐dwarf plant stature, which is regarded as a favourable trait in cereal cultivation (Sakamoto et al., 2006; Ahmar & Gruszka, 2023), without the negative effect on plant fertility.

Interestingly, the second substitution identified in our study ‐ G449D (the *hvdwf5.1k* allele) did not cause any significant alteration in height, tillering, and spike length of the identified homozygous mutants. Our further analysis indicated that although the substituted amino acid residue is highly conserved among homologous proteins from monocot and dicot species (Figure S5), the G449D substitution did not affect the presence of both sterol reductase functional domains within the encoded enzyme (the substituted residue is localised outside of these functional domains). These results may explain the lack of impact of this mutation on the above‐mentioned aspects of phenotype of the identified *hvdwf5.1k* mutant.

Apart from the missense mutations, this study identified the T791C substitution within the donor site of the intron 2 (allele *hvdwf5.j*). The homozygous mutants carrying this allele showed semi‐dwarf phenotype, however, they remained fully fertile. Thus, it is another proof that mutation in the *HvDWF5* gene in barley may allow for the development of semi‐dwarf plant architecture without the side effect on plant fertility. Taking into account the phenotype of the *hvdwf5.j* mutant, it was hypothesized that it may result from abnormal splicing of the intron 2 from the *HvDWF5* transcript. Indeed, the RT‐PCR analysis indicated that the *hvdwf5.j* mutation results in retention of the intron 2 within transcripts extracted from roots and leaves of 8‐day‐old seedlings of the *hvdwf5.j* mutant. Interestingly, retention of this intron was also observed in the *HvDWF5* transcript variant extracted from leaves of 8‐day‐old ‘Sebastian’ seedlings (Figure 4B). It may indicate that the *HvDWF5* transcript is alternatively spliced in leaves of the ‘Sebastian’ seedlings. In fact, we have retrieved information from the Ensembl Plants database indicating that the *HvDWF5* gene (HORVU.MOREX.r3.3HG0320030) has 4 splice variants, whereas in the EoRNA database, 13 splice variants of the *HvDWF5* gene have been reported. Expression profiles of the *HvDWF5* transcript variants are shown in Figure 5 and Figure S3. Importantly, each splice variant is characterised by different expression levels and spatial expression patterns. These data may explain the intron retention observed in the *HvDWF5* transcript extracted from ‘Sebastian’ leaves and the additional amplicons obtained in the RT‐PCR reaction. Noteworthy, the alternative splicing has never been observed in any transcript encoded by a gene involved in the BR biosynthesis. Taking into account that intron retention is the most frequent outcome of alternative splicing in plants and the splice variants with retained intron(s) take part in plant development and responses to environmental stresses (Wong et al., 2016), this organ‐specific retention of the second intron within the *HvDWF5* transcript may have an important role in plant adaptation. Noteworthy, although the intron retention causes the 96‐bp‐long insertion in the coding sequence of the transcript, our further analysis showed that it does not cause a frameshift, as the reading frame of the transcript is not disrupted (in‐frame insertion), and the 32‐amino‐acid‐long fragment is incorporated into the mutant version of the HvDWF5 protein (Figure 7A and C). However, this structural change of the encoded protein affects its function, as it was reported that the brassinolide accumulation was significantly reduced in the *hvdwf5.j* mutant (Figure 6). Despite the lack of influence of the retained second intron on the presence of the sterol reductase domains in the HvDWF5 protein in roots and leaves of the *hvdwf5.j* mutant (Figure 7A, C and Table 2), its semi‐dwarf phenotype may stem from the production of the new version of the enzyme, which shows altered localisation of the sterol reductase (functional) domains and distribution of transmembrane (structural) domains (Table S4) and/or undergoes altered post‐translational modifications (Wong et al., 2016; Wong & Schmitz, 2022).

This study also identified the G5246A substitution in the donor site of the intron 12 (allele *hvdwf5.1i*). In this case, it was necessary to analyse the *HvDWF5* gene sequence in 112 individual plants constituting progeny of heterozygous M_2_ plant carrying this mutation. Out of this group of plants, a single homozygous *hvdwf5.1i* mutant was identified. The height of the *hvdwf5.1i* mutant was significantly reduced (dwarfism) and constituted only 31% of the height of the cultivar ‘Sebastian’. Moreover, the *hvdwf5.1i* mutant showed complete sterility (Figure 2). Thus, taking into account the severe mutant phenotype, we postulated that it may be caused by a defect in splicing of the intron 12 from the *HvDWF5* transcript. The RT‐PCR analysis indicated that the *hvdwf5.1i* mutation results in retention of the intron 12 within the *HvDWF5* transcript (Figure 4C). This intron retention causes the frameshift mutation, which results in the substitution of 9 consecutive amino acids, disruption of the second sterol reductase domain, and additionally, premature stop codon leading to truncation of the encoded protein (Figure 7A, D and Table 2). Thus, it is another example of the correlation between the severity of the mutant phenotype (including a decrease in fertility) and the impact of mutations on the activity of the sterol reductase domains. Noteworthy, the *dwf5‐2* and *dwf5‐6* mutants of Arabidopsis in which mutations affected the 3′ splice site of the intron 8 and 5′ splice site of the intron 12, respectively, and resulted in premature stop codons also led to a significant reduction of plant fertility, exemplified by reduced seed set (Choe et al., 2000). Further research also confirmed that plant sterols play a crucial role in the regulation of organogenesis and the development of reproductive organs (Du et al., 2022).

In our study, both intron retention mutations (alleles *hvdwf5.j* and *hvdwf5.1i*) caused some changes in the arrangement of secondary structural domains within the encoded versions of the HvDWF5 protein (Figure S4). However, our analyses with the ScanProsite and InterPro programs indicated that the *hvdwf5.j* mutation led to changes (relative to the version of the HvDWF5 protein in the ‘Sebastian’ cultivar) in the positions of both sterol reductase functional domains, although the sequence and length of each domain in this mutant remained intact. Importantly, according to the analysis, this mutation did not abolish the function of these sterol reductase domains (Figure 7A, C and Table 2). On the contrary, the *hvdwf5.1i* mutation resulted in the lack of a functional second sterol reductase domain (Figure 7A, D and Table 2). This difference in the number of functional sterol reductase domains between the *hvdwf5.j* and *hvdwf5.1i* mutants may underlie the diversity in the observed severity of their phenotypes ‐ semi‐dwarfism and fertility (*hvdwf5.j*) vs. dwarfism and sterility (*hvdwf5.1i*). Indeed, two *dwf5* mutants of Arabidopsis (*dwf5‐2* and *dwf5‐3*), which displayed the most severe phenotypes, were devoid of the second sterol reductase domain due to intron retention resulting in a premature stop codon (*dwf5‐2*) and nonsense mutation (*dwf5‐3*) (Choe et al., 2000). These results reported in the *dwf5* mutants in Arabidopsis seem to confirm the conclusions reached in our study.

## 5 CONCLUSIONS AND PERSPECTIVES

This study provided a novel insight into the role of the *HvDWF5* gene in the BR biosynthesis‐dependent regulation of architecture and reproduction of barley as an important cereal species. Moreover, some of the barley mutants identified in this study showed semi‐dwarfism, being of particular importance for cereal breeding and yield, however, without any negative effect on grain size and weight, which was previously observed in other BR mutants of cereals. It indicated that mutations of this gene allow for a balance between the favourable alteration in plant architecture (semi‐dwarfism) and maintenance of grain size in this important crop. It was indicated by application of various approaches (including analysis of BR‐deficient and BR‐insensitive mutants) that BRs regulate plant tolerance to various environmental stresses, including drought (Gruszka et al., 2016a; Fabregas et al., 2018; Gruszka et al., 2020), low and high temperature (Pociecha et al., 2020; Sadura et al., 2020), salinity (Li et al., 2020), heavy metals (Rajewska et al., 2016; Kour et al., 2021), and pathogen attack (Ali et al., 2014; Nawaz et al., 2017). It was indicated in the several above‐mentioned reports that the BR‐deficient and BR‐insensitive (semi‐)dwarf mutants may show enhanced tolerance to these environmental stresses. Moreover, an accumulating body of evidence indicates that genetic modifications or biotechnological manipulations (gene editing, overexpression, silencing or miRNA‐mediated regulation) of the BR homeostasis in cereals may facilitate the development of germplasm with improved tolerance to various environmental challenges, such as drought, salinity, oxidative stress, thermal stress, and biotic stresses (Zolkiewicz & Gruszka, 2025). Therefore, the semi‐dwarf barley mutants described in this study may be regarded as promising input into future breeding experiments to develop new germplasm with improved tolerance to environmental stresses.

## AUTHOR CONTRIBUTIONS

KZ: conceptualization, methodology, investigation, data curation, visualization, writing ‐ original draft, writing ‐ review & editing; JO: methodology, investigation; BC: methodology, investigation; DG: conceptualization, methodology, formal analysis, investigation, data curation, visualization, writing ‐ original draft, writing ‐ review & editing, supervision, funding acquisition.

## FUNDING INFORMATION

This work was supported by the National Science Centre, Poland [grant No. 2019/35/B/NZ2/00382].

## CONFLICT OF INTEREST STATEMENT

No conflict of interest declared.

## Supporting information


**Table S1.** Primer pairs (DWF5_1F_1R, DWF5_2F_2R, DWF5_3F_3R, DWF5_4F_4R) applied for amplification of fragments of the *HvDWF5* genomic sequence. The primer pairs DWF5_Ex2‐3, DWF5_Ex9‐10, DWF5_Ex10‐11, DWF5_Ex12‐13 were used for amplification of the *HvDWF5* transcript fragments containing the intron 2, intron 9, intron 10 and intron 12, respectively.
**Table S2.** PCR profiles for the DWF5_2F_2R, DWF5_3F_3R, DWF5_1F_1R and DWF5_4F_4R primers.
**Table S3.** RT‐PCR profile for the DWF5_Ex2‐3, DWF5_Ex9‐10, DWF5_Ex10‐11, DWF5_Ex12‐13 primers.
**Table S4.** Impact of the identified mutations *hvdwf5.1 h*, *hvdwf5.j* and *hvdwf5.1i* on the distribution of transmembrane domains in the HvDWF5 protein, predicted with the use of DeepTHMHMM tool.
**Figure S1.** Exemplary homozygous mutant lines carrying mutations which did not have significant impact on plant phenotype in comparison with the reference cultivar ‘Sebastian’. Plants of each of the genotypes are presented at the same developmental stage. Scale bar = 10 cm.
**Figure S2.** Histological analysis of vascular bundles distribution based on transverse sections of the second (from the bottom) internodes of the *hvdwf5.1i* mutant and the reference cultivar ‘Sebastian’. Vascular bundles are indicated by white arrows. The internode samples were collected from mature plants of each genotype and stained with calcofluor. Scale bar: 100 μm.
**Figure S3.** Expression profile analysis of the *HvDWF5* transcript variants (TPM ‐ transcript per million).
**Figure S4.** Impact of the identified mutations (*hvdwf5.1 h*, *hvdwf5.1i* and *hvdwf5.j*) on the secondary structure of the encoded versions of the HvDWF5 protein, predicted by the PSIPRED tool.
**Figure S5.** The multiple sequence alignment (MSA) of the DWF5 proteins with the use of Clustal Omega tool. The positions of substituted amino acids (R422K, allele *hvdwf5.1 h*; G449D, allele *hvdwf5.1 k*) as a result of the identified mutations are indicated by black frames. Hela *‐ Helianthus annuus*, Arat *‐ Arabidopsis thaliana*, Brad *‐ Brachypodium distachyon*, Orys *‐ Oryza sativa*, Zeam *‐ Zea mays*, Sorb *‐ Sorghum bicolor*, Horv *‐ Hordeum vulgare*, Tria *‐ Triticum aestivum*, Aegt *‐ Aegilops tauschii*.
**Table S5.** Positions of amino acid residues which participate in binding various ligands by the HvDWF5 protein. The R422 residue was substituted in the *hvdwf5.1 h* mutant. The D423 residue participates in binding the NADP molecule. Both positions are highlighted in red. C‐score [0–1] is the confidence score of the prediction.

## Data Availability

Sequence of the *HvDWF5* gene (HORVU.MOREX.r3.3HG0320030) was retrieved from the Ensembl Plants database at https://plants.ensembl.org/index.html. All primary data to support the findings of this study regarding the transcript variants of the *HvDWF5* gene are openly available in the Ensembl Plants database at https://plants.ensembl.org/index.html (Yates et al. 2022) and the EoRNA database at https://ics.hutton.ac.uk/eorna/index.html (Milne et al., 2021). The germplasm described in the article will be available from the corresponding author upon reasonable request. All data generated or analysed during this study are included in this article and its supplementary information files (Data self‐contained within the manuscript and in the Supporting information).
