# Supplementary material for: Mutations of the brassinosteroid biosynthesis gene HvDWARF5 enable balance between semi‐dwarfism and maintenance of grain size in barley
Source: Physiol Plant. 2025 Mar 24;177(2):e70179. doi: 10.1111/ppl.70179 (PMC11933512; doi:10.1111/ppl.70179)
Supplement: Supplementary file 1 — Table S1. Primer pairs (DWF5_1F_1R, DWF5_2F_2R, DWF5_3F_3R, DWF5_4F_4R) applied for amplification of fragments of the HvDWF5 genomic sequence. The primer pairs DWF5_Ex2‐3, DWF5_Ex9‐10, DWF5_Ex10‐11, DWF5_Ex12‐13 were used for amplification of the HvDWF5 transcript fragments containing the intron 2, intron 9, intron 10 and intron 12, respectively. Table S2. PCR profiles for the DWF5_2F_2R, DWF5_3F_3R, DWF5_1F_1R and DWF5_4F_4R primers. Table S3. RT‐PCR profile for the DWF5_Ex2‐3, DWF5_Ex9‐10, DWF5_Ex10‐11, DWF5_Ex12‐13 primers. Table S4. Impact of the identified mutations hvdwf5.1 h, hvdwf5.j and hvdwf5.1i on the distribution of transmembrane domains in the HvDWF5 protein, predicted with the use of DeepTHMHMM tool. Figure S1. Exemplary homozygous mutant lines carrying mutations which did not have significant impact on plant phenotype in comparison with the reference cultivar ‘Sebastian’. Plants of each of the genotypes are presented at the same developmental stage. Scale bar = 10 cm. Figure S2. Histological analysis of vascular bundles distribution based on transverse sections of the second (from the bottom) internodes of the hvdwf5.1i mutant and the reference cultivar ‘Sebastian’. Vascular bundles are indicated by white arrows. The internode samples were collected from mature plants of each genotype and stained with calcofluor. Scale bar: 100 μm. Figure S3. Expression profile analysis of the HvDWF5 transcript variants (TPM ‐ transcript per million). Figure S4. Impact of the identified mutations (hvdwf5.1 h, hvdwf5.1i and hvdwf5.j) on the secondary structure of the encoded versions of the HvDWF5 protein, predicted by the PSIPRED tool. Figure S5. The multiple sequence alignment (MSA) of the DWF5 proteins with the use of Clustal Omega tool. The positions of substituted amino acids (R422K, allele hvdwf5.1 h; G449D, allele hvdwf5.1 k) as a result of the identified mutations are indicated by black frames. Hela ‐ Helianthus annuus, Arat ‐ Arabidopsis thaliana, Brad ‐ Brach [file PPL-177-e70179-s001.pdf]

Table S1. Primer pairs (DWF5_1F_1R, DWF5_2F_2R, DWF5_3F_3R, DWF5_4F_4R) applied for amplification of fragments of the *HvDWF5* genomic sequence. The primer pairs DWF5_Ex2-3, DWF5_Ex9-10, DWF5_Ex10-11, DWF5_Ex12-13 were used for amplification of the *HvDWF5* transcript fragments containing the intron 2, intron 9, intron 10 and intron 12, respectively.

Primer Name	Primer Sequence	GC%	Tm (°C)
**DWF5_1F_1R**	F: 5'CACAGATCCACACATCCTCCT 3'	52,3	64,1
	R: 5' AGACCTGGGGCAACATACTG 3'	55	63,9
**DWF5_2F_2R**	F: 5' TGCGAATGCTATGGACAAAC 3'	45	63,6
	R: 5' TCCGTTGTTTCTTCTAACACAGA 3'	39,1	62,5
**DWF5_3F_3R**	F: 5' TTTGTCTGACATAATTTTCAACAGC 3'	32	62,9
	R: 5'GCTACATATGACTGGAGGATTGTTT 3'	40	63,1
**DWF5_4F_4R**	F: 5' CGTCACTTTCACTATGCCCC 3'	55	64,4
	R: 5'ATACAAGCTACCACTGCACG 3'	50	60,4
**DWF5_Ex2-3**	F: 5' GCAGATCTACGAGCACCTCC 3'	60	55,9
	R: 5'AGGTGAGCAAAGTCACTGCAT 3'	48	52,4
**DWF5_Ex9-10**	F: 5' CTCTCGATTCTCCTTGCTGG 3'	55	53,8
	R: 5' CTGGTTTTTGTTTCTCCCTTTG 3'	41	51,1
**DWF5_Ex10-11**	F: 5'ACAAAGGGAGAAACAAAAACCA 3'	36	49,2
	R: 5' GGATCTCTGGGGCATAGTGA 3'	55	53,8
**DWF5_Ex12-13**	F: 5' TACTGCTTGACCGAGCGAAG 3'	55	53,8
	R: 5' CCAGGAACAACCCTGTAAGGTA 3'	50	54,8

Table S2. PCR profiles for the DWF5_2F_2R, DWF5_3F_3R, DWF5_1F_1R and DWF5_4F_4R primers.

	Temperature [°C]		Duration	No. of cycles
	**DWF5_2F_2R** and **DWF5_3F_3R**	**DWF5_1F_1R** and **DWF5_4F_4R**		
Initial denaturation	95°C	95°C	5 mins	1
Denaturation	95°C	95°C	45 sec	
Annealing	64°C	68°C	50 sec	3
Extension	72°C	72°C	1 min 30 sec	
Denaturation	95°C	95°C	45 sec	
Annealing	62°C	66°C	50 sec	3
Extension	72°C	72°C	1 min 30 sec	
Denaturation	95°C	95°C	45 sec	
Annealing	60°C	64°C	50 sec	36
Extension	72°C	72°C	1 min 30 sec	
Final extension	72°C	72°C	5 mins	1
Pause	15°C	15°C	Pause	1

Table S3. RT-PCR profile for the DWF5_Ex2-3, DWF5_Ex9-10, DWF5_Ex10-11, DWF5_Ex12-13 primers.

	Temperature [°C]				Duration	No. of cycles
	**DWF5_Ex2-3**	**DWF5_Ex9-10**	**DWF5_Ex10-11**	**DWF5_Ex12-13**		1
Initial denaturation	95°C	95°C	95°C	95°C	3 mins	1
Denaturation	95°C	95°C	95°C	95°C	45 sec	
Annealing	63°C	63°C	63°C	63°C	45 sec	4
Extension	72°C	72°C	72°C	72°C	1 min	
Denaturation	95°C	95°C	95°C	95°C	45 sec	
Annealing	61°C	61°C	61°C	61°C	45 sec	4
Extension	72°C	72°C	72°C	72°C	1 min	
Denaturation	95°C	95°C	95°C	95°C	45 sec	
Annealing	59,4°C	58,8°C	58,2°C	60°C	45 sec	36
Extension	72°C	72°C	72°C	72°C	1 min	
Final extension	72°C	72°C	72°C	72°C	5 mins	1
Pause	8°C	8°C	8°C	8°C	Pause	1

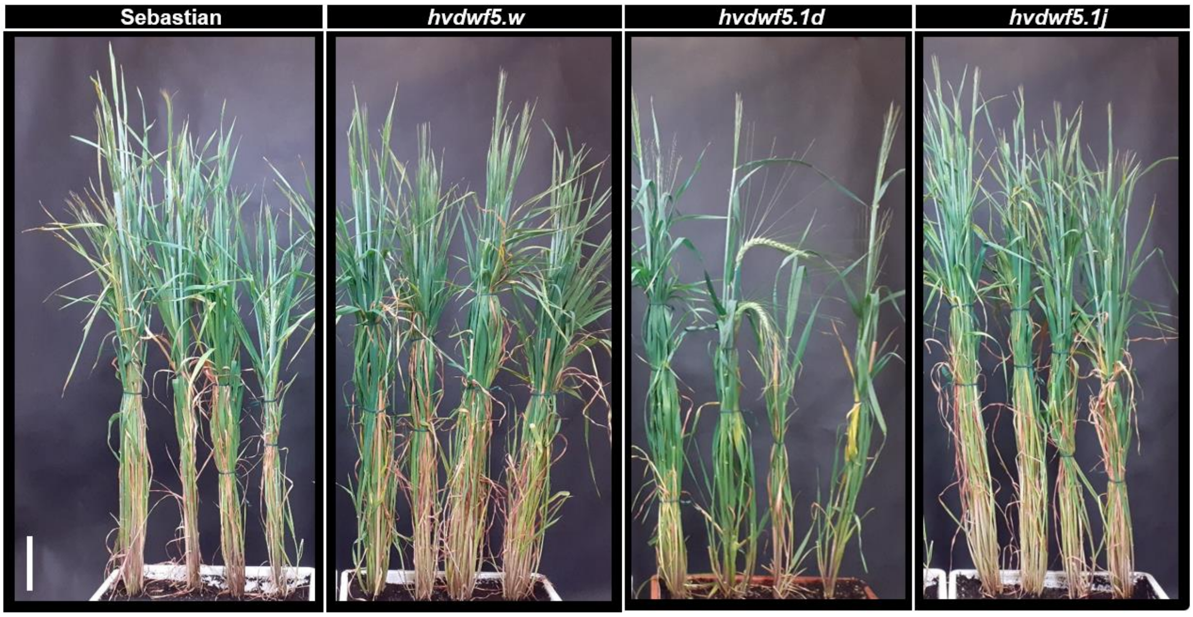

Fig. S1. Exemplary homozygous mutant lines carrying mutations which did not have significant impact on plant phenotype in comparison with the reference cultivar ‘Sebastian’. Plants of each of the genotypes are presented at the same developmental stage. Scale bar = 10 cm.

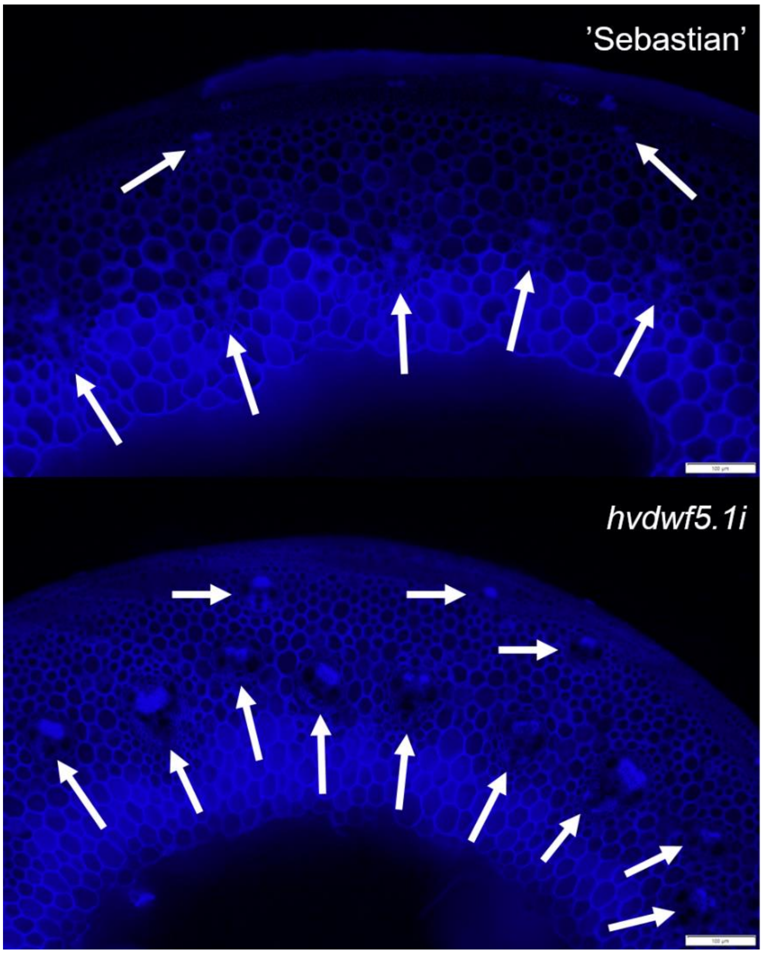

Fig. S2. Histological analysis of vascular bundles distribution based on transverse sections of the second (from the bottom) internodes of the *hvdwf5.1i* mutant and the reference cultivar ‘Sebastian’. Vascular bundles are indicated by white arrows. The internode samples were collected from mature plants of each genotype and stained with calcofluor. Scale bar: 100 μm.

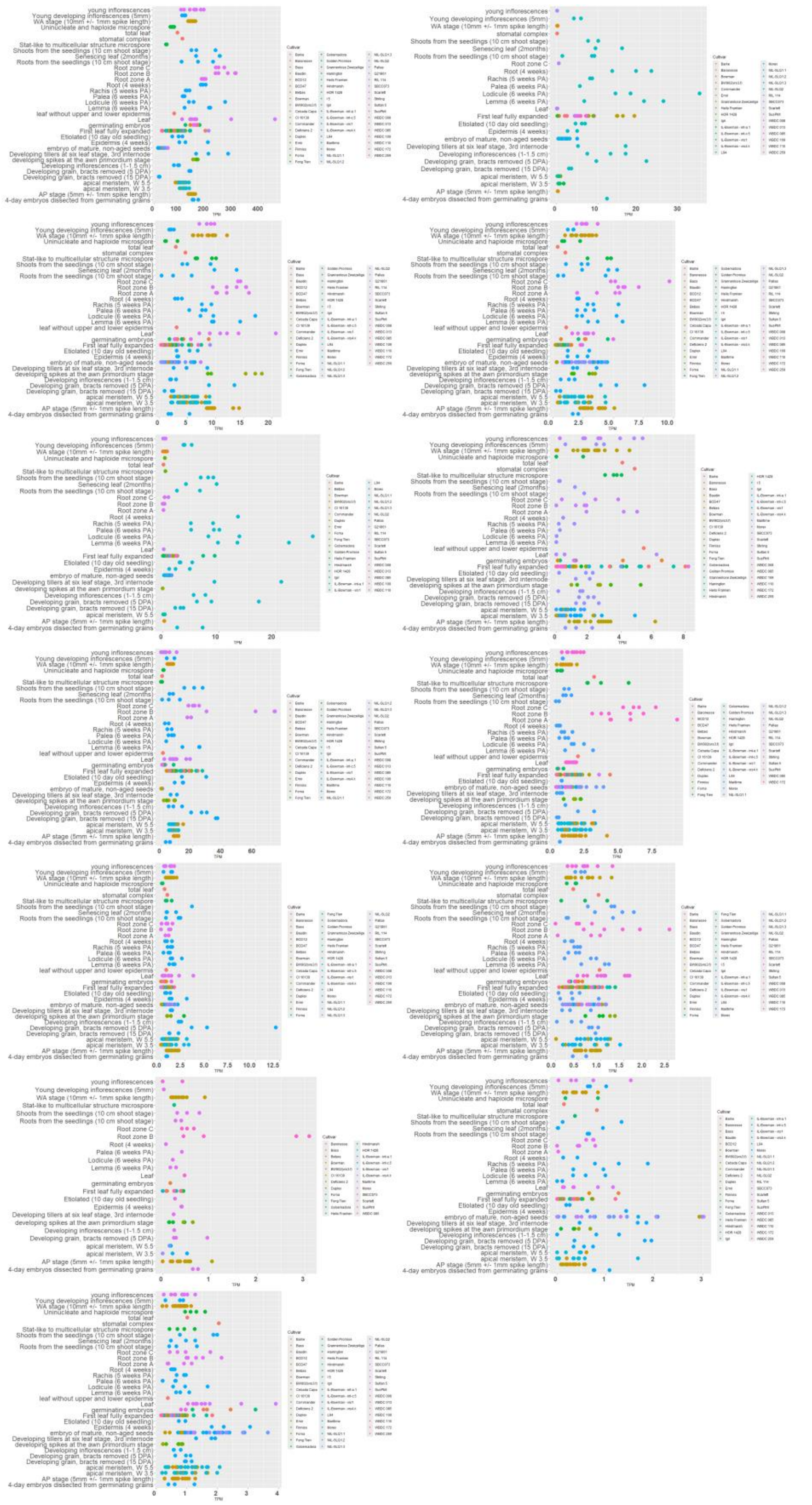

Fig. S3. Expression profile analysis of the *HvDWF5* transcript variants (TPM - transcript per million). The expression data for these transcripts in various tissues were retrieved from the EoRNA database.

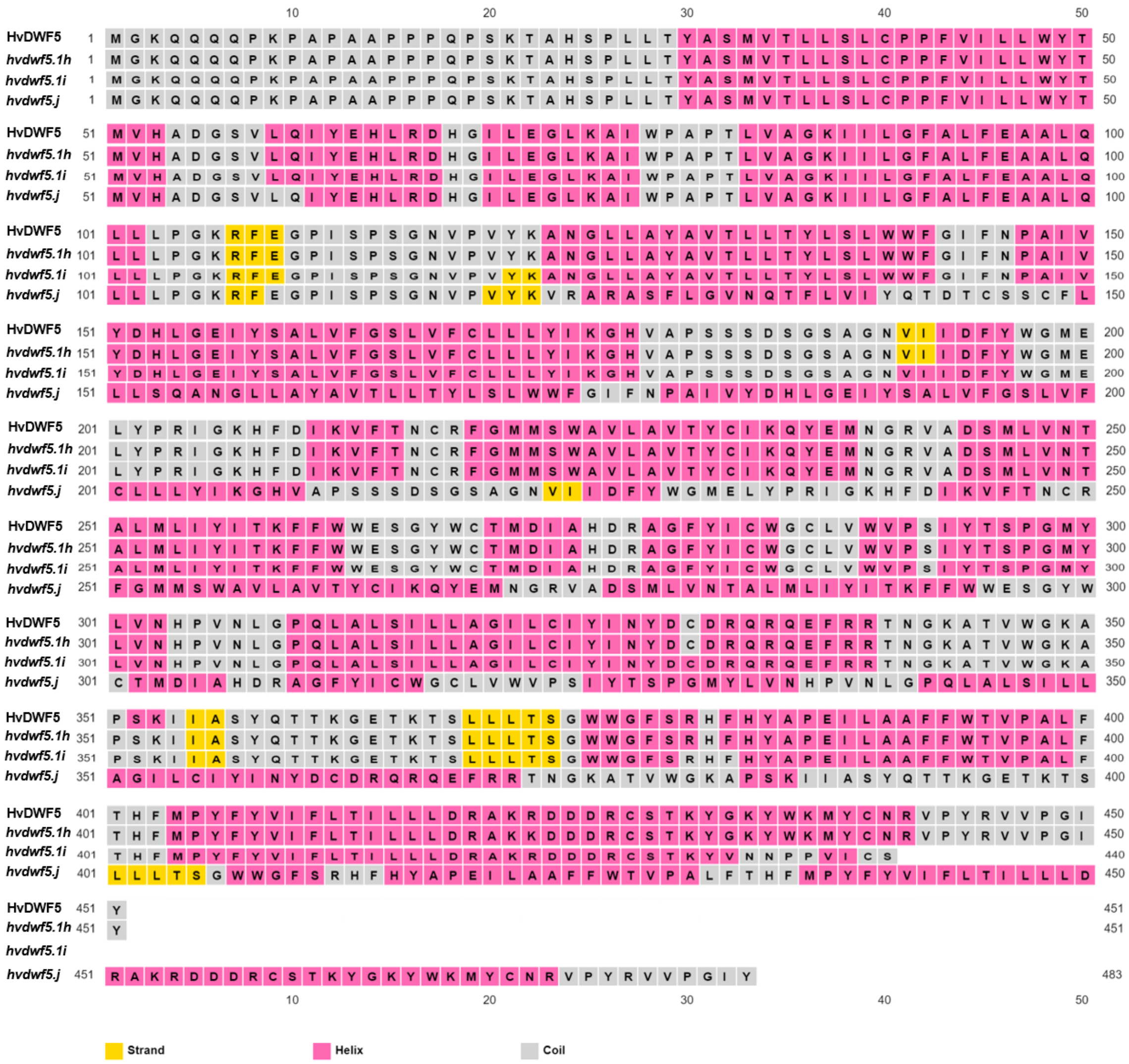

Fig. S4. Impact of the identified mutations (*hvdwf5.1h*, *hvdwf5.1i* and *hvdwf5.j*) on the secondary structure of the encoded versions of the HvDWF5 protein, predicted by the PSIPRED tool.

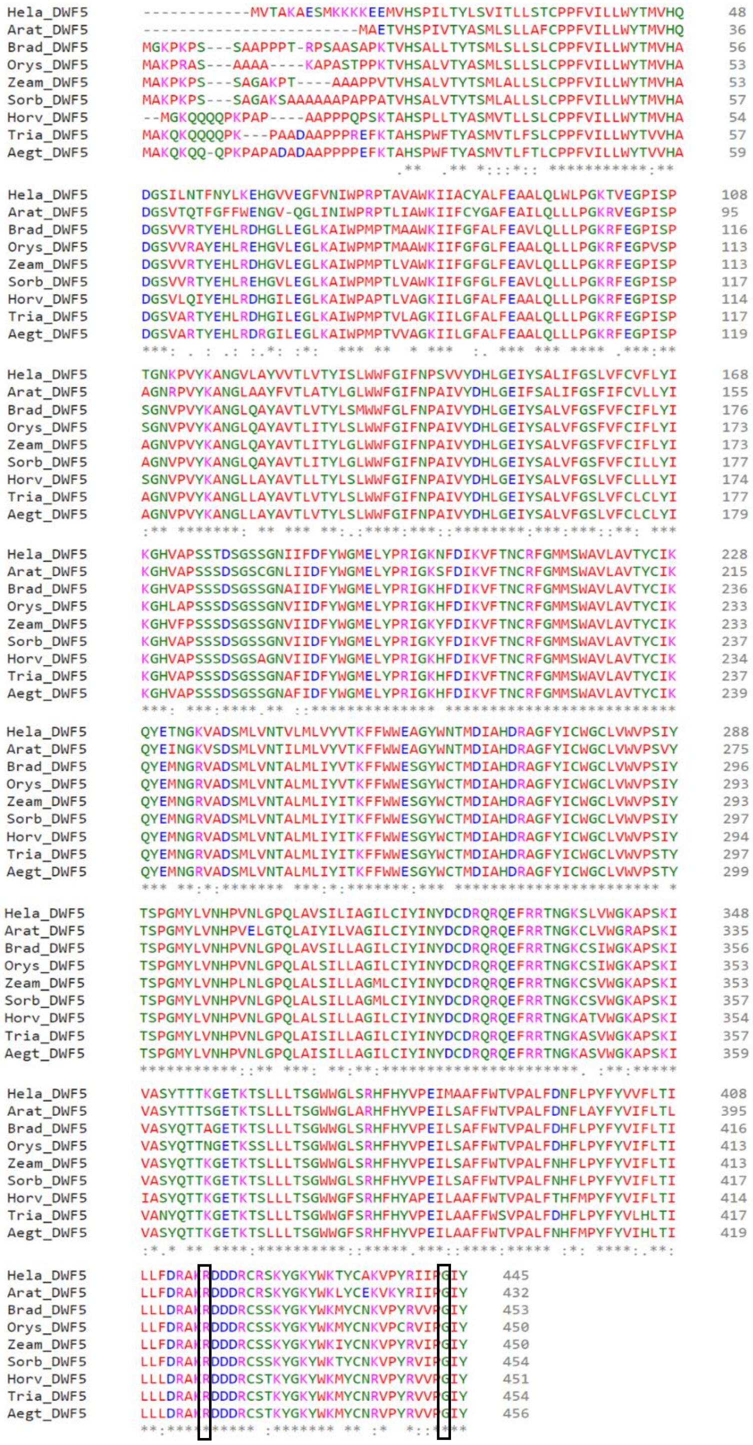

Fig. S5. The multiple sequence alignment (MSA) of the DWF5 proteins with the use of Clustal Omega tool. The positions of substituted amino acids (R422K, allele *hvdwf5.1h*; G449D, allele *hvdwf5.1k*) as a result of the identified mutations are indicated by black frames. Hela - *Helianthus annuus*, Arat - *Arabidopsis thaliana*, Brad - *Brachypodium distachyon*, Orys - *Oryza sativa*, Zeam - *Zea mays*, Sorb - *Sorghum bicolor*, Horv - *Hordeum vulgare*, Tria - *Triticum aestivum*, Aegt - *Aegilops tauschii*.

Table S4. Impact of the identified mutations *hvdwf5.1h*, *hvdwf5.j* and *hvdwf5.1i* on the distribution of transmembrane domains in the HvDWF5 protein, predicted with the use of DeepTHMHMM tool.

	**WT**	***hvdwf5.1h***	***hvdwf5.j***	***hvdwf5.1i***
Number of predicted TMRs	10	10	10	10
Inside	1-32	1-32	1-32	1-32
TMhelix	33-51	33-49	33-51	33-48
Outside	52-85	50-85	52-87	49-85
TMhelix	86-104	86-104	88-103	86-104
Inside	105-123	105-123	104-156	105-123
TMhelix	124-138	124-138	157-170	124-138
Outside	139-156	139-156	171-188	139-156
TMhelix	157-174	157-174	189-206	157-174
Inside	175-212	175-212	207-244	175-212
TMhelix	213-233	213-233	245-265	213-233
Outside	234-246	234-246	266-278	234-246
TMhelix	247-264	247-264	279-296	247-264
Inside	265-278	265-278	297-310	265-278
TMhelix	279-299	279-299	311-331	279-299
Outside	300-310	300-310	332-342	300-310
TMhelix	311-328	311-328	343-360	311-327
Inside	329-384	329-384	361-416	328-384
TMhelix	385-394	385-394	417-426	385-394
Outside	395-396	395-396	427-428	395-396
TMhelix	397-417	397-417	429-449	397-417
Inside	418-451	418-451	450-483	418-440

>Sebastian_*HvDWF5*_CDS (length: 1356 bp)

ATGGGGAAGCAGCAGCAGCAGCCCAAGCCGGCCCCCGCGGCGCCGCCGCCCCAGCCGTCCAA
GACGGCGCACTCGCCGTTGTTAACCTACGCCTCCATGGTCACGCTCTTGTCCCTCTGCCCGC
CCTTCGTCATCCTCCTGTGGTACACCATGGTGCACGCGGACGGATCGGTGCTGCAGATCTAC
GAGCACCTCCGCGACCACGGGATCCTGGAGGGGCTCAAGGCCATCTGGCCCGCGCCCACCCT
CGTCGCCGGGAAGATCATCCTCGGCTTCGCTCTCTTCGAGGCCGCCCTGCAGCTGCTCCTCC
CCGGGAAGCGCTTCGAGGGGCCCATCTCGCCCTCCGGCAATGTGCCCGTCTACAAGGCAAAT
GGTTTACTAGCATATGCAGTGACTTTGCTCACCTACCTAAGCCTCTGGTGGTTTGGAATTTT
TAACCCTGCGATAGTGTATGATCACCTGGGAGAGATATACTCTGCTCTGGTCTTTGGAAGCC
TTGTGTTCTGTTTATTGCTGTACATAAAGGGCCATGTAGCACCATCTTCATCTGATTCCGGA
TCCGCAGGGAATGTGATAATTGATTTCTACTGGGGAATGGAACTATATCCGCGGATTGGTAA
GCACTTCGATATCAAAGTCTTCACAAACTGCCGTTTTGGGATGATGTCCTGGGCTGTTCTTG
CTGTAACCTACTGCATAAAGCAGTATGAAATGAATGGCCGAGTTGCAGACTCCATGCTTGTG
AATACTGCACTGATGTTGATCTATATCACAAAGTTTTTCTGGTGGGAGTCTGGATATTGGTG
TACTATGGACATTGCACATGATAGAGCTGGTTTCTACATTTGCTGGGGATGCTTGGTATGGG
TTCCATCAATTTACACCTCTCCTGGAATGTACCTTGTCAATCATCCTGTGAATTTGGGTCCC
CAGCTAGCACTCTCGATTCTCCTTGCTGGAATATTGTGCATATACATAAACTATGACTGTGA
TCGTCAGCGCCAAGAATTTCGCCGGACAAACGGGAAAGCCACAGTCTGGGGCAAAGCCCCGT
CAAAGATTATTGCTTCCTATCAGACAACAAAGGGAGAAACAAAAACCAGTCTTCTCTTGACT
TCTGGATGGTGGGGCTTTTCTCGTCACTTTCACTATGCCCCAGAGATCCTAGCAGCATTTTT
CTGGACCGTTCCAGCTCTTTTCACTCATTTTATGCCATACTTCTATGTGATATTTCTGACCA
TTCTACTGCTTGACCGAGCGAAGAGGGATGATGATAGATGCTCAACAAAGTACGGCAAGTAC
TGGAAGATGTACTGCAACAGAGTACCTTACAGGGTTGTTCCTGGTATTTACTGA

>*hvdwf5.j_HvDWF5*_CDS (length: 1452 bp)

ATGGGGAAGCAGCAGCAGCAGCCCAAGCCGGCCCCCGCGGCGCCGCCGCCCCAGCCGTCCAA
GACGGCGCACTCGCCGTTGTTAACCTACGCCTCCATGGTCACGCTCTTGTCCCTCTGCCCGC
CCTTCGTCATCCTCCTGTGGTACACCATGGTGCACGCGGACGGATCGGTGCTGCAGATCTAC
GAGCACCTCCGCGACCACGGGATCCTGGAGGGGCTCAAGGCCATCTGGCCCGCGCCCACCCT
CGTCGCCGGGAAGATCATCCTCGGCTTCGCTCTCTTCGAGGCCGCCCTGCAGCTGCTCCTCC
CCGGGAAGCGCTTCGAGGGGCCCATCTCGCCCTCCGGCAATGTGCCCGTCTACAAGGTCCGT
GCCCGTGCTTCATTCCTGGGAGTCAACCAGACATTTCTGGTGATATATCAGACAGATACCTG
CAGCTCATGCTTTCTTCTTTTGTCTCAGGCAAATGGTTTACTAGCATATGCAGTGACTTTGC
TCACCTACCTAAGCCTCTGGTGGTTTGGAATTTTTAACCCTGCGATAGTGTATGATCACCTG
GGAGAGATATACTCTGCTCTGGTCTTTGGAAGCCTTGTGTTCTGTTTATTGCTGTACATAAA
GGGCCATGTAGCACCATCTTCATCTGATTCCGGATCCGCAGGGAATGTGATAATTGATTTCT
ACTGGGGAATGGAACTATATCCGCGGATTGGTAAGCACTTCGATATCAAAGTCTTCACAAAC
TGCCGTTTTGGGATGATGTCCTGGGCTGTTCTTGCTGTAACCTACTGCATAAAGCAGTATGA
AATGAATGGCCGAGTTGCAGACTCCATGCTTGTGAATACTGCACTGATGTTGATCTATATCA
CAAAGTTTTTCTGGTGGGAGTCTGGATATTGGTGTACTATGGACATTGCACATGATAGAGCT
GGTTTCTACATTTGCTGGGGATGCTTGGTATGGGTTCCATCAATTTACACCTCTCCTGGAAT
GTACCTTGTCAATCATCCTGTGAATTTGGGTCCCCAGCTAGCACTCTCGATTCTCCTTGCTG
GAATATTGTGCATATACATAAACTATGACTGTGATCGTCAGCGCCAAGAATTTCGCCGGACA
AACGGGAAAGCCACAGTCTGGGGCAAAGCCCCGTCAAAGATTATTGCTTCCTATCAGACAAC
AAAGGGAGAAACAAAAACCAGTCTTCTCTTGACTTCTGGATGGTGGGGCTTTTCTCGTCACT
TTCACTATGCCCCAGAGATCCTAGCAGCATTTTTCTGGACCGTTCCAGCTCTTTTCACTCAT
TTTATGCCATACTTCTATGTGATATTTCTGACCATTCTACTGCTTGACCGAGCGAAGAGGGA

TGATGATAGATGCTCAACAAAGTACGGCAAGTACTGGAAGATGTACTGCAACAGAGTACCTT
ACAGGGTTGTTCCTGGTATTTACTGA

>*hvdwf5.1i_HvDWF5*_CDS (length: 1500 bp)

ATGGGGAAGCAGCAGCAGCAGCCCAAGCCGGCCCCCGCGGCGCCGCCGCCCCAGCCGTCCAA
GACGGCGCACTCGCCGTTGTTAACCTACGCCTCCATGGTCACGCTCTTGTCCCTCTGCCCGC
CCTTCGTCATCCTCCTGTGGTACACCATGGTGCACGCGGACGGATCGGTGCTGCAGATCTAC
GAGCACCTCCGCGACCACGGGATCCTGGAGGGGCTCAAGGCCATCTGGCCCGCGCCCACCCT
CGTCGCCGGGAAGATCATCCTCGGCTTCGCTCTCTTCGAGGCCGCCCTGCAGCTGCTCCTCC
CCGGGAAGCGCTTCGAGGGGCCCATCTCGCCCTCCGGCAATGTGCCCGTCTACAAGGCAAAT
GGTTTACTAGCATATGCAGTGACTTTGCTCACCTACCTAAGCCTCTGGTGGTTTGGAATTTT
TAACCCTGCGATAGTGTATGATCACCTGGGAGAGATATACTCTGCTCTGGTCTTTGGAAGCC
TTGTGTTCTGTTTATTGCTGTACATAAAGGGCCATGTAGCACCATCTTCATCTGATTCCGGA
TCCGCAGGGAATGTGATAATTGATTTCTACTGGGGAATGGAACTATATCCGCGGATTGGTAA
GCACTTCGATATCAAAGTCTTCACAAACTGCCGTTTTGGGATGATGTCCTGGGCTGTTCTTG
CTGTAACCTACTGCATAAAGCAGTATGAAATGAATGGCCGAGTTGCAGACTCCATGCTTGTG
AATACTGCACTGATGTTGATCTATATCACAAAGTTTTTCTGGTGGGAGTCTGGATATTGGTG
TACTATGGACATTGCACATGATAGAGCTGGTTTCTACATTTGCTGGGGATGCTTGGTATGGG
TTCCATCAATTTACACCTCTCCTGGAATGTACCTTGTCAATCATCCTGTGAATTTGGGTCCC
CAGCTAGCACTCTCGATTCTCCTTGCTGGAATATTGTGCATATACATAAACTATGACTGTGA
TCGTCAGCGCCAAGAATTTCGCCGGACAAACGGGAAAGCCACAGTCTGGGGCAAAGCCCCGT
CAAAGATTATTGCTTCCTATCAGACAACAAAGGGAGAAACAAAAACCAGTCTTCTCTTGACT
TCTGGATGGTGGGGCTTTTCTCGTCACTTTCACTATGCCCCAGAGATCCTAGCAGCATTTTT
CTGGACCGTTCCAGCTCTTTTCACTCATTTTATGCCATACTTCTATGTGATATTTCTGACCA
TTCTACTGCTTGACCGAGCGAAGAGGGATGATGATAGATGCTCAACAAAGTATGTAAACAAT
CCTCCAGTCATATGTAGC**TAA**CATTGACAGAGGAACTTATCTTTAAATTGCTCGGACGCTTA
TTTGGTTAGTTTTCAGTTCTCTGGTTCTGTCCTTGATAGATTGCTCTTTTCTTTCTTGCTCT
CTGAAAGGTACGGCAAGTACTGGAAGATGTACTGCAACAGAGTACCTTACAGGGTTGTTCCT
GGTATTTACTGA

Fig. S6. Coding sequences (CDS) of the full length *HvDWF5* transcript variants from the cultivar ‘Sebastian’ and the *hvdwf5.j* and *hvdwf5.1i* mutants. Sequences of the 2nd and 12th intron retained within the *HvDWF5* transcript variants produced by the *hvdwf5.j* and *hvdwf5.1i* mutants, respectively, as highlighted in red. Premature stop codon introduced into the coding sequence as a consequence of the frameshift mutation in the *hvdwf5.1i* is shown in black frame.

Table S5. Positions of amino acid residues which participate in binding various ligands by the HvDWF5 protein. The R422 residue was substituted in the *hvdwf5.1h* mutant. The D423 residue participates in binding the NADP molecule. Both positions are highlighted in red. C-score [0-1] is the confidence score of the prediction.

	**Ligand name**	**Ligand full name**	**Ligand Binding Site Residues**	**C-score**
**1**	NDP	NADPH DIHYDRO-NICOTINAMIDE-ADENINE-DINUCLEOTIDE PHOSPHATE	269,331,334,338,369,370,371, 376,381,383,384,423,427,430, 431,435,438	0.27
**2**	ALA	ALANINE	227,295	0.08
**3**	N/A	?	216,221,267,270,272,276,285,294 ,334,370,376,381,383,384,387, 408,412,416,419,422,423,426, 427,431	0.07
**4**	DXC	DEOXYCHOLIC ACID	319,322,323,326,386,389	0.05
**5**	VAL	VALINE	392,409	0.05
